# Neuronal Death Mechanisms and Therapeutic Strategy in Ischemic Stroke

**DOI:** 10.1007/s12264-022-00859-0

**Published:** 2022-05-05

**Authors:** Rui Mao, Ningning Zong, Yujie Hu, Ying Chen, Yun Xu

**Affiliations:** 1 Department of Neurology, Affiliated Drum Tower Hospital, Medical School of Nanjing University, Nanjing, 210008 China; 2 The State Key Laboratory of Pharmaceutical Biotechnology, Institute of Brain Science, Nanjing University, Nanjing, 210008 China; 3 Jiangsu Key Laboratory for Molecular Medicine, Medical School of Nanjing University, Nanjing, 210008 China; 4 Jiangsu Province Stroke Center for Diagnosis and Therapy, Nanjing, 210008 China; 5 Nanjing Neurology Clinic Medical Center, Nanjing, 210008 China

**Keywords:** Ischemic stroke, Neuronal death, Mechanisms, Therapeutic strategy

## Abstract

Ischemic stroke caused by intracranial vascular occlusion has become increasingly prevalent with considerable mortality and disability, which gravely burdens the global economy. Current relatively effective clinical treatments are limited to intravenous alteplase and thrombectomy. Even so, patients still benefit little due to the short therapeutic window and the risk of ischemia/reperfusion injury. It is therefore urgent to figure out the neuronal death mechanisms following ischemic stroke in order to develop new neuroprotective strategies. Regarding the pathogenesis, multiple pathological events trigger the activation of cell death pathways. Particular attention should be devoted to excitotoxicity, oxidative stress, and inflammatory responses. Thus, in this article, we first review the principal mechanisms underlying neuronal death mediated by these significant events, such as intrinsic and extrinsic apoptosis, ferroptosis, parthanatos, pyroptosis, necroptosis, and autophagic cell death. Then, we further discuss the possibility of interventions targeting these pathological events and summarize the present pharmacological achievements.

## Introduction

Globally, stroke is among the primary causes of mortality and disability [1]. Among strokes, ischemic stroke (IS) accounts for >70%. Neuronal death caused by cerebral ischemia determines the mortality and disability rate of stroke. In general, reversing neuronal death in the ischemic infarct core is very challenging, and for many years, strategies that have focused on preventing neuronal death in the penumbra (the region surrounding the infarct core) and secondary neuronal loss have failed because of unclear mechanisms.

Multiple paradigms of cell death have been progressively defined since the last century. Aside from the commonly-known forms of cell death, such as apoptosis, neurons can undergo several rarely known modes, such as ferroptosis, pyroptosis, and parthanatos [2]. Any kind of cell death is inseparable from a cascade of detrimental events that occur in IS, especially in the pathological processes of excitotoxicity, oxidative stress, and inflammatory responses. These deleterious pathological events are mutually independent and extensively intersect in promoting cell death.

**Fig. 1:**
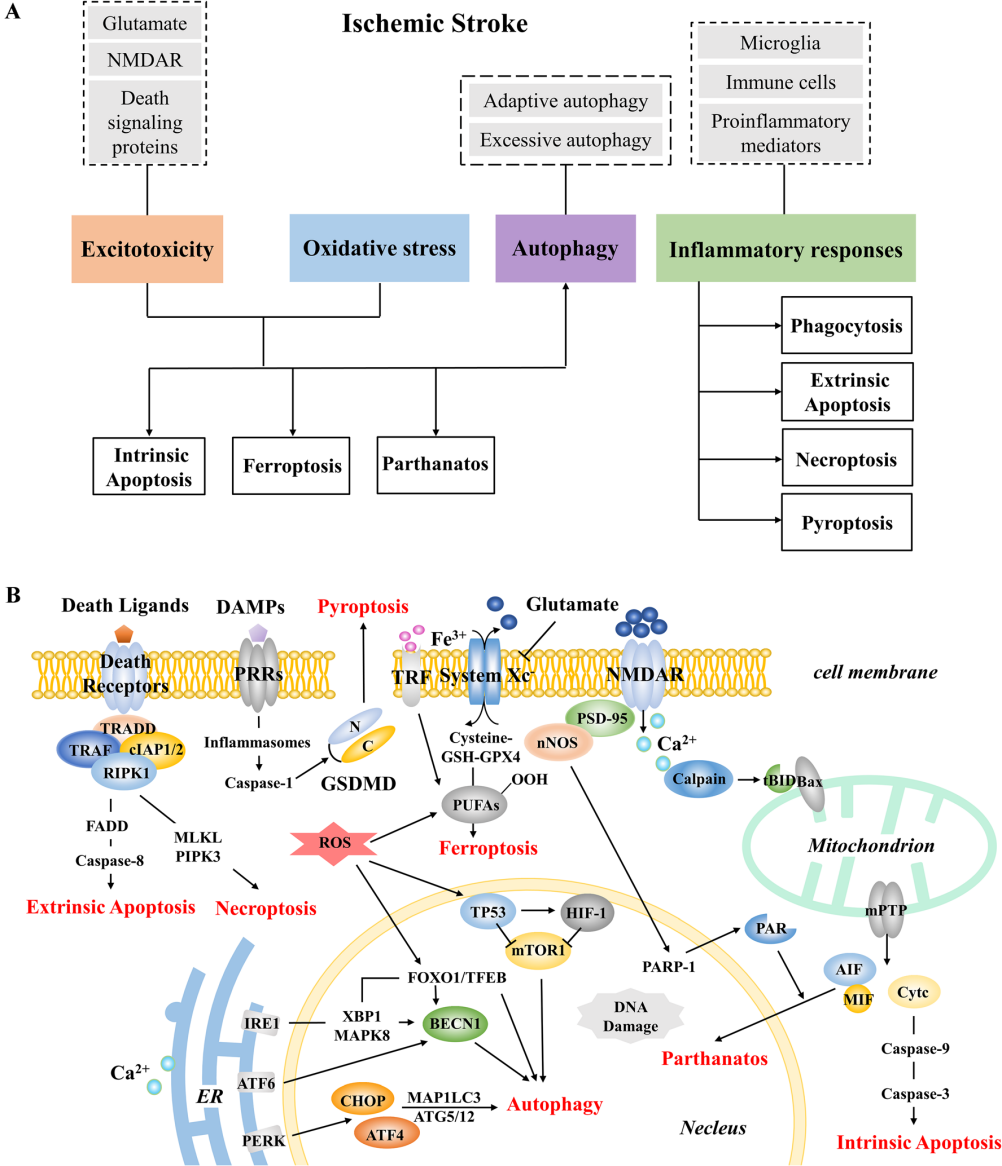
**A** The main structure of this review. The content in the dashed frame represents the relevant therapeutic targets. **B** A simple schematic of several death mechanisms in ischemic stroke.

Here, we review the mechanisms of the significant events following IS and the different forms of neuronal death secondary to these pathological factors. We also illustrate the therapeutics of excitotoxicity, oxidative stress, and inflammatory responses, in order to enrich the currently limited clinical treatment. Considering the particularity of autophagy, we list this separately for discussion (Fig. 1).

## Excitotoxicity and Neuronal Death

As the primary neurotransmitter in the central nervous system (CNS), glutamate is responsible for rapid synaptic transmission so that communication among neurons can be realized. But it has neurotoxic effects under pathological conditions. This special type of neurotoxicity mediated by excitatory amino-acids is called excitotoxicity, which is the key link between ischemia and neuronal death in strokes.

Among the currently-known ionotropic and metabotropic glutamate receptors, N-methyl-d-aspartate receptors (NMDARs) play an important role in permitting excessive Ca^2+^ influx, which in turn leads to ischemic cell death. Growing evidence has demonstrated the dual effects of NMDARs on neuronal outcomes [3], which can be boiled down to several hypotheses, such as the ‘NMDAR subtype’ hypothesis and the ‘NMDAR location’ hypothesis.

Overactivated NMDARs during cerebral ischemia are heterotetrameric complexes involving two essential NR1 (GluN1) subunits and two NR2 (GluN2) subunits [4]. The ‘NMDAR subtype’ hypothesis emphasizes that GluN2AR conduces to neuronal survival while GluN2BR induces neuronal death [5]. In addition, as the second hypothesis suggests, synaptic NMDARs contribute to neuronal survival while extrasynaptic receptors activate distinct downstream death signaling proteins [6]. But multiple studies have demonstrated that both synaptic and extrasynaptic NMDARs participate in triggering the cell death signaling pathway [3].

The pro-survival effects of NMDARs are attributable to activation of the PI3K (phosphoinositide-3-kinase)–Akt (protein kinase B) pathway, activation of the ERK (extracellular signal regulated kinase) pathway, and expression of the CREB (cAMP-response element binding protein)-related gene in an activity-dependent manner.

It is worth noting that NMDARs mediate neuronal death or survival according to their activity. Hyper-activity or hypo-activity is often detrimental, whereas normal NMDAR activity promotes cell survival. Both high concentrations of NMDA and strong pharmacological inhibition induce neuronal death. Research has revealed the dynamic changes of NMDAR activity in the acute neurodestructive phase and the subsequent recovery phase of IS [6, 7]. A late-onset persistent decline of NMDAR activity has been reported after overactivation. This can prevent secondary neuronal loss in the penumbra and increase neurogenesis in the dentate gyrus [8].

### Excitotoxicity and Apoptosis by the Intrinsic/Mitochondrial Pathway

In 1972, Kerr *et al.* first described the morphological features of apoptosis, including nuclear and cytoplasmic condensation, apoptotic body formation, and cell fragmentation [9]. As is widely known, apoptotic cell death is programmed and can be initiated by the intrinsic (mitochondrial) or the extrinsic (death receptor) pathway. Ca^2+^ overload mediated by excitotoxicity during cerebral ischemia leads to apoptosis principally through the former pathway.

Increased intracellular Ca^2+^ mainly depends on rapid Ca^2+^ influx *via* NMDARs and other non-excitotoxic means, including acid‐sensing ion channels [10], transient receptor potential channels [11], and Na^+^/Ca^2+^ exchangers [12]. The dysfunction of these homeostasis regulators gradually causes Ca^2+^ overload, hence triggering calpain activation [13]. Calpain can cleave Bcl‐2 interacting domain (BID) to produce tBID, which interacts with Bax (Bcl-2-associated X protein). Bax forms homo-oligomers and then inserts them into the mitochondrial outer membrane. As a result, mitochondrial permeability transition pores take shape [2, 14, 15] and thus allow the release of apoptogens.

**Fig. 2:**
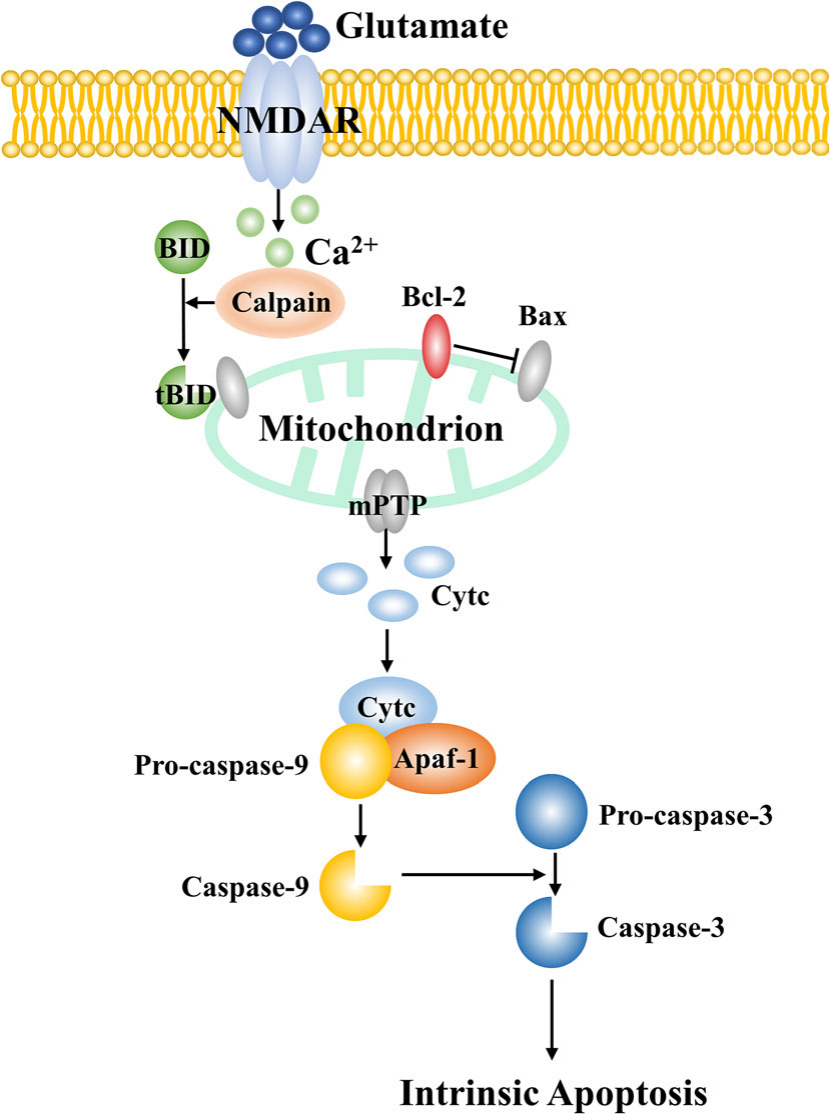
Apoptosis by the intrinsic/mitochondrial pathway during cerebral ischemia. Increased extracellular glutamate activates NMDARs, causing excessive Ca^2+^ influx. Activated calpain cleaves BID to tBID, which interacts with Bax and helps to form the mPTP. Cytc is released from these pores and combines with Apaf-1 and pro-caspase-9. The apoptosome stepwise activates the executioner caspase-3, and consequently induces apoptosis. Apaf‐1, apoptotic protein‐activating factor‐1; Bcl‐2, B‐cell leukemia/lymphoma 2; BID, Bcl-2 interacting domain; Cytc, cytochrome C; mPTP, mitochondrial permeability transition pore; NMDA, N‐methyl‐d‐aspartate; tBID, truncated Bid.

Cytochrome C (Cytc) is one of the most important pro-apoptotic factors. Upon entering the cytosol, Cytc combines with apoptotic protein-activating factor‐1 (Apaf-1) and procaspase‐9 to form the apoptosome. Procaspase-9 depends on autocleavage to become mature, thereby progressively activating the executor caspase-3 to mediate intrinsic apoptosis [16, 17] (Fig. 2).

In addition, when neurons undergo ischemic insults, NMDARs can activate death-associated protein kinase 1 (DAPK1) and promote DAPK1-p53-dependent apoptotic cell death [18]. Phosphatase and tensin homolog deleted on chromosome TEN (PTEN) dephosphorylates PIP3 into PIP2, inhibiting the PI3K–Akt survival signaling pathway [19]. GluN2BR-mediated PTEN nuclear translocation has also been shown to induce apoptosis [20]. C-Jun N-terminal kinase (JNK) is also involved in caspase-dependent apoptosis by inducing the phosphorylation of Bcl-2-associated death promoter [21].

### Excitotoxicity and Ferroptosis

Ferroptosis, a lately discovered form of non-apoptotic death, was first defined in 2012. It is iron-dependent and is fundamentally attributable to the overwhelming lipid peroxidation. From a morphological point of view, ferroptosis with mitochondrial shrinkage can be distinguished from other forms of cell death [22, 23]. There is evidence that ferroptosis may be triggered by excitotoxic stress when neurons are exposed to hypoxia.

The overactivation of NMDARs following stroke can induce iron uptake through the NMDAR-Dexras1-PAP7- divalent metal transporter 1 (DMT1) signaling cascade, leading to increased redox-active iron in the neuronal cytosol [24, 25]. Apart from intracellular iron deposition, the reduction of the cysteine-GSH-GPX4 axis activity promotes ferroptosis as well [26]. During excitotoxic stress, the extravagant accumulation of glutamate inhibits the cystine/glutamate antiporter, System Xc^−^, which reduces the influx of cystine and limits the biosynthesis of GSH. GPX4, the key negative regulator of ferroptosis, uses GSH to catalyze lipid hydroperoxides into alcohols [27]. Hence, low levels of GSH can weaken the activity of GPX4 and promote overwhelming peroxidation.

**Fig. 3:**
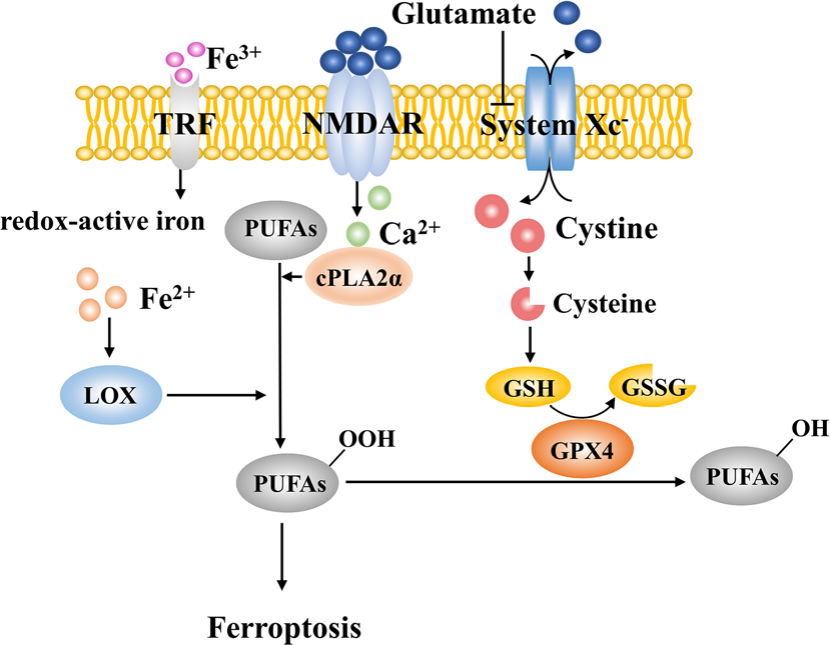
Ferroptosis during cerebral ischemia. Normally, GPX4 uses GSH to catalyze lipid hydroperoxides into alcohols. Increased extracellular glutamate inhibits the activity of System Xc^−^ as well as the cysteine-GSH-GPX4 axis. Meanwhile, Ca^2+^ overload activates cPLA2α to provide substrates for lipid peroxidation. Fe^2+^-activated lipoxygenase (LOX) also participates in this process. Increased redox-active iron and overwhelming lipid peroxidation contribute to ferroptosis. cPLA2α, cytosolic phospholipase A2α; GPX4, γ‐L‐glutamyl‐L‐cysteinylglycine peroxidase 4; GSH, glutathione; GSSG, oxidized glutathione; LOX, lipoxygenase; PUFA, polyunsaturated fatty acid; TRF, transferrin.

Ca^2+^ overload can activate cytosolic phospholipase A2α (cPLA2α), which mediates the release of arachidonic acid and lysophospholipid to provide substrates for lipid peroxidation [28]. All the mechanisms described above can work together to eventually trigger ferroptotic neuronal death in IS [14]. Many studies have reported that ferroptosis inhibitors including Ferrostatin-1 and Liproxstatin-1 prevent glutamate-induced neurotoxicity and have neuroprotection effects [26, 29] (Fig. 3).

### Excitotoxicity and Parthanatos

Poly (ADP-ribose) polymerase-1 (PARP-1) is a nuclear protein that limits genomic instability and facilitates DNA base repair as well as the regulation of inflammatory processes [30]. Nevertheless, in many neurodegenerative diseases, rapid PARP-1 activation can result in a unique cell death mode called parthanatos [31, 32].

During cerebral ischemia, the C-terminal domains of GluN2B binding to postsynaptic density protein‐95 (PSD-95) [33] link NMDARs to downstream neurotoxic molecules such as nitric oxide synthase (nNOS) [34]. In addition to PSD-95, PSD-93 is another molecular adaptive protein that glues NMDAR and nNOS together [35]. The GluN2B-PSD95/93-nNOS complex combines excitotoxicity with neuronal DNA injury that is caused by oxidative or nitrosative stress. When DNA damage occurs, PARP-1 can use NAD^+^ as a substrate to synthesize PAR, thereby inducing the nuclear translocation of apoptosis-inducing factor (AIF) [36–38].

**Fig. 4:**
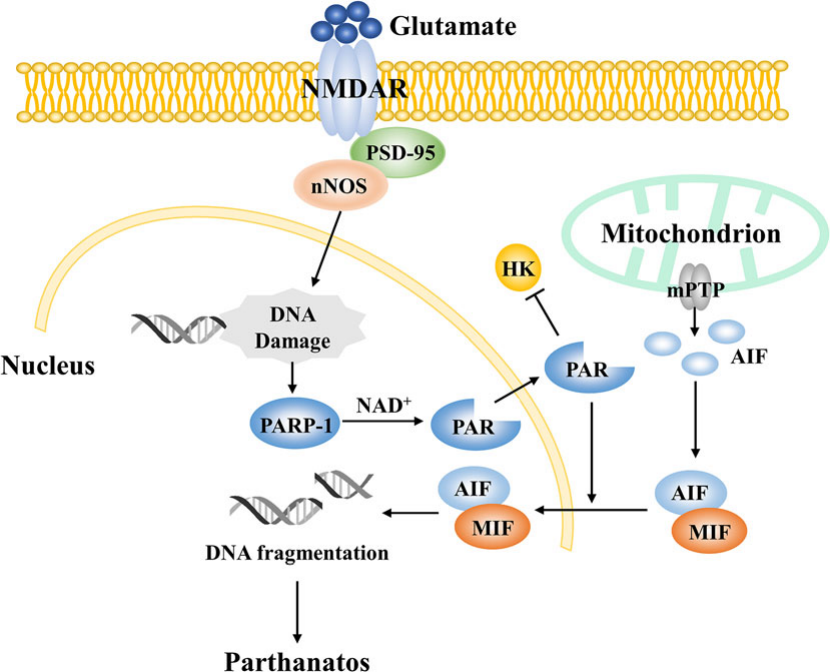
Parthanatos during cerebral ischemia. PSD-95 links NMDARs to nNOS. Neuronal DNA injury that is caused by oxidative or nitrosative stress activates PARP-1, which uses NAD^+^ to synthesize PAR. PAR inhibits HK and promotes the nuclear translocation of AIF. The mPTP allows the release of AIF into the cytoplasm where it binds to MIF. The AIF–MIF complex enters the nucleus and mediates DNA fragmentation. HK, hexokinase; MIF, migration inhibitory factor; NAD^+^, nicotinamide adenine dinucleotide; nNOS, nitric oxide synthase; PAR, poly (ADP-ribose); PARP-1, poly (ADP-ribose) polymerase-1; PSD-95, postsynaptic density protein‐95.

The formation of mPTPs allows the release of AIF into the neuronal cytosol during excitotoxic stress. During this process, AIF recruits migration inhibitory factor (MIF) and they both rapidly translocate to the nucleus, which eventually mediates DNA fragmentation and chromatin condensation [39]. Besides, PAR promotes bioenergetic collapse and causes neuronal death by inhibiting hexokinase, one of the key rate-limiting enzymes of glycolysis [40]. At the same time, NAD^+^ depletion impairs metabolic processes, causes mitochondrial dysfunction, and aggravates cellular damage [36] (Fig. 4).

Excitotoxicity also induces autophagic cell death in IS.

### Therapeutics of Excitotoxicity in Ischemic Stroke

#### Targeting Glutamate

Previous preclinical research has shown that decreasing the release or enhancing the reuptake of glutamate are effective therapeutic candidates against ischemic injury. However, the majority of these established agents are inefficient in reducing morbidity or mortality in stroke clinical trials, and have more or less neurological side-effects. Currently, there are several potential therapeutic targets worth exploration. For instance, the latest research found that the subunit Swell1 of the volume-regulated anion channel activated by cell swelling is responsible for the release of glutamate [41]. N-myc downstream regulated gene 2 interacts with Na^+^/K^+^-ATPase β1 to facilitate glutamate uptake in astrocytes [42].

Compared with drug treatment, blood glutamate scavenging does not interfere with normal brain neurophysiology [43]. Peritoneal dialysis has been found effective in reducing blood glutamate in rats during cerebral ischemia [44]. This finding needs to be further verified in clinical trials.

#### Targeting NMDARs

As early as the late 1980s, researchers discovered that the NMDAR antagonist dizocilpine (MK-801) significantly protects the brain against focal cerebral ischemia in the rat [45]. With an in-depth understanding of their pharmacological effects, NMDAR antagonists can be divided into competitive antagonists at the recognition site, uncompetitive ion channel blockers, and subunit-specific antagonists [46]. A series of drugs have been reported to be effective against ischemic insults both *in vitro* and *in vivo*, such as uncompetitive NMDAR antagonists (ketamine, phencyclidine, dextromethorphan, and dizocilpine) and glycine-binding site antagonists (gavestinel and licostine). Nevertheless, there is a lack of clinical success regarding the fact that these agents may induce pathomorphological changes and neurological dysfunction [5].

In recent years, based on the ‘NMDAR subtype’ hypothesis, more and more targeted therapies and interventions have entered the preclinical stage. GluN2BR-selective NMDA antagonists, like CP-101 606 and Ro 25-6981 [47, 48], failed in clinical trials due to safety reasons and limited efficacy, even though they were effective against neuronal damage. Similar drugs still face the same problems, including a short therapeutic window and serious side-effects [3]. Along with progress in the reduction of adverse effects, the pH-dependent GluN2BR-selective antagonists, the 93-series, may be more clinically promising because they have less impact on the healthy brain at normal pH [49]. A novel synthesized brain-penetrant GluN2BR antagonist called compound 45e also exhibits superior neuroprotective activity [50]. By contrast, some current studies prefer to exploit GluN2AR-positive allosteric modulators to enhance neuronal survival signaling [51, 52].

Although the validity of the ‘NMDAR location’ hypothesis is still under discussion, interventions based on it have already attained certain achievements. Memantine, which is used to treat Alzheimer’s disease, has been found to preferentially block the open channels of extrasynaptic NMDARs at therapeutic concentrations. Its pharmacological effects on neuroprotection and neuroplasticity have been confirmed in preclinical strokes [53, 54].

#### Targeting Downstream Death Signaling Proteins

DAPK1 activation is inseparable from calcineurin activation. It is through reducing calcineurin activity that Protopanaxadiol ginsenoside-Rd alleviates the DAPK1-mediated phosphorylation of GluN2B [55]. Besides, Caytaxin also inhibits DAPK1 catalytic activity. It combines with DAPK1 at the presynaptic site and kicks in 2 h after middle cerebral artery occlusion (MCAO) [56]. Likewise, the interference peptide Tat-NR2B-CT blocks the effects of DAPK1 on GluN2B [57].

The PTEN inhibitor bpv, which is a bisperoxovanadium compound, decreases MCAO-induced neuronal apoptosis by preventing the downregulation of phospho-mTOR. Therefore, it can be concluded that PTEN deletion protects the ischemic brain by activating the mTOR survival signaling pathway [58]. Moreover, a considerable number of microRNAs prevent cerebral ischemic damage through the PTEN-PI3K-Akt pathway, including miR-130a [59], miR-532-5p [60], miR-188-5p [61], and miR-217-5p [62]*.*

JNK-IN-8, a highly specific JNK inhibitor, suppresses the JNK/NF-κB pathway and the activation of microglia. It decreases the expression of several pro-inflammatory factors and further controls neuroinflammation as well as ischemic injury [63]. Roflumilast, approved for the treatment of chronic obstructive pulmonary disease, has been found to prevent neurons from ischemia/reperfusion injury by attenuating the phosphorylation of JNK [64]. Regrettably, none of the above agents have achieved clinical application for the treatment of IS.

Tat-NR2B-9c (NA-1) is an interference peptide that binds to PSD-95. The neuroprotective effect of NA-1 against ischemic insults has been demonstrated in rodent models and non-human primate models [65]. It is encouraging that the phase III clinical trial, ESCAPE-NA1, achieved significant results. This trial enrolled 1,105 patients who suffered acute IS within 12 h to measure the efficacy and safety of NA-1 [66]. According to the results, there are still problems to be solved with respect to the timing and strategy of NA-1 though its value cannot be denied. TP95 is a cell-penetrating peptide containing PSD-95 cleavage sites. When delivered to the cortex, it downregulates PSD-95 and improves the neurological outcome during excitotoxic stress [67].

## Oxidative Stress and Neuronal Death

When an IS occurs, neurons generate excessive amounts of superoxide compounds, including hydroxyl radical and nitric oxide, due to hypoxia and subsequent reperfusion stimulation. The generation of these molecules is related to nicotinamide adenine dinucleotide phosphate (NADPH) oxidase, cyclooxygenases, and xanthine oxidase [68, 69], among which, NADPH oxidase is considered to be the major source of reactive oxygen species (ROS) in the brain.

As an electron donor, NADPH produces O_2_^−^ in response to NADPH oxidase (NOX) catalysis. NOX is a membrane-bound enzyme complex consisting of seven subtypes that have been detected in neuronal cells, microglia, fibroblasts, and endothelial cells [70]. After the occurrence of IS, NOX2, NOX1, NOX4, and NOX5 in neuronal cells are activated to produce peroxides such as H_2_O_2_ and O_2_- [68]. NOX has been studied from the perspective of the nervous system and it was found that the expression of NOX2 and NOX4 are increased in neuronal cells in IS. Besides, through negatively regulating NOX2, miR-320 reduces the generation of ROS in IS so as to protect neurons [71].

The continuous accumulation of oxidative molecules in cells leads to the peroxidation of lipids and the cross-linking of macromolecules such as DNA and proteins. These oxidative molecules also mediate signaling pathways which induce different types of cell death [72], such as ferroptosis, apoptosis, and autophagy.

### Oxidative Stress and Apoptosis

After a stroke, the depolarization of the cell membrane caused by ATP depletion is accompanied by the massive production of ROS. ROS can directly damage the integrity of plasma membrane and can lead to DNA strand breaks as well. Besides, it seems to participate in the release of Cytc from mitochondria into the cytoplasm of neurons. A research team also found that ultraviolet irradiation or anticancer drugs generate ROS, which then mediate apoptosis by activating apoptosis signal-regulating kinase 1 [73]. In brief, the damage caused by ROS promotes apoptosis in many ways [14].

Oxidative stress primarily damages mitochondria, including mitochondrial DNA, Ca^2+^ ion balance, and the mitochondrial membrane, and induces apoptosis by the intrinsic pathway. The key element of apoptosis in the mitochondrial pathway is mitochondrial outer membrane permeabilization, which can be regulated by affecting the Bcl-2 protein family or downstream caspase [73, 74].

Under oxidative stress, as a nuclear transcription factor, p53 is activated and then regulates pro-apoptotic genes such as Bax and Bak (Bcl-2 antagonist or killer). It also directly acts on mitochondria, resulting in the release of Cytc and the activation of caspase, and finally induces apoptosis [75]. It has been shown that oxidative stress responsive apoptosis inducing protein acts on the MAPK signaling pathway and the JAK/STAT signaling pathway to activate caspase, causing caspase-dependent apoptosis [76].

In addition, studies have shown that TRPM2, which belongs to the transient receptor potential channel superfamily, is associated with the normal transport of Ca^2+^ and is highly sensitive to oxidative stress. When stimulated by oxidative stress, TRPM2 channels cause the destruction of intracellular Ca^2+^ and Zn^2+^ homeostasis. The increasing intracellular Ca^2+^ concentration plays an important role in initiating apoptosis [14, 77].

### Oxidative Stress and Ferroptosis

Furthermore, iron overload and excessive lipid peroxidation are closely linked to oxidative stress and contribute to ferroptosis.

Normally, under the gradual action of transferrin and DMT1, the iron in a cell is continuously transformed in the state of Fe^2+^ and Fe^3+^, and circulates throughout the body, and is eventually transported to the labile iron pool (LIP) in the cytoplasm in the form of Fe^2+^ [78]. The iron level in the LIP is transferred by ferritin (FT), and regulated by both FT and iron response protein (IRP), to keep the Fe^2+^ concentration in the LIP in a narrow range to achieve dynamic balance [79]. When cells are under oxidative stress, ROS and NOS act on IRP1, IRP2, and other proteins, leading to intracellular iron overload by regulating the IRP/iron response element system [80, 81]. Consequently, excessive iron damages cells through the Fenton reaction and lipid peroxidation. During cerebral ischemia, the accumulating iron in cells enters the brain parenchyma through the damaged blood-brain barrier [78]. Excessive Fe^2+^ combines with a large number of superoxides in the brain to produce Fe^3+^ and hydroxyl radical. At the same time, Fe^2+^ is an active molecule in the catalytic subunit of LOX, which catalyzes lipid peroxidation. The overproduced hydroxyl radical and lipid peroxide induce oxidative stress and cell death [82].

Lipid peroxidation also provides abundant ROS for the oxidative stress of neuronal cells. Fatty acids in the brain stem account for about half of the mass, of which polyunsaturated fatty acid (PUFA) accounts for ~40%. PUFA often contains multiple double bonds, among which arachidonic acid and adrenergic acid are particularly susceptible to oxidization to form lipid peroxidation products [83, 84]. The production of lipid peroxides results in a massive accumulation of ROS and lipid hydrogen peroxide. And the amount and location of PUFA are related to the degree of lipid peroxidation, which indirectly determines the degree of the ferroptosis effect [85].

GSH and oxidized glutathione (GSSG) constitute the antioxidant system in cells. This system eliminates oxidative species to protect cells from oxidative damage. Previous studies have shown that there is a certain relationship between cell oxidative death and the significant depletion of GSH/GSSG, and have also shown that the inducer used in the test consumes GSH by inhibiting system Xc^−^ [86]. At the same time, GSH combines with Fe^2+^ in the LIP, so GSH directly prevents Fe^2+^ from oxidizing to generate hydroxyl radical and reduce the damage of cells caused by oxidative stress [85]. Sesamin reduces the ischemic brain injury in mice by reducing the levels of lipid peroxidation and superoxide anion and restoring the level of GSH [87].

Oxidative stress also participates in facilitating autophagy.

### Therapeutics of Oxidative Stress in Ischemic Stroke

Here is a brief list of some novel drugs with therapeutic potential. (1) Prussian blue (PB) has been used clinically as an antidote to metal poisoning such as by cesium, and as a complex contrast agent for ultrasound. New studies have found that PB has the same internal enzyme activity as Fe_3_O_4_ to protect cells from oxidative stress and plays an anti-apoptotic role by eliminating ROS and adjusting the expression of p53 and Bcl-2 in cells [88]. (2) Calycosin-7-O-β-D-glucoside is the main component of *Astragalus* isoflavones. It plays a protective role in neurons after IS through decreasing SOD activity, apoptosis rate, and Bax protein expression, and activating the STRTI/FoxO1/PGC-1α signaling pathway [89]. (3) Melatonin, which is already used to regulate sleep and delays aging, has antioxidation and anti-autophagy effects by blocking NF-κB signal transduction and activating the mTOR signaling pathway in experimental stroke [90]. (4) Chlorogenic acid is a polyphenol. It protects the brain against stroke by activating the Nrf2 signaling pathway, which enhances the activity of SOD and GSH, and reduces the content of intracellular ROS and the rate of apoptosis [91]. (5) Carvacrol, a phenolic monoterpenoid that targets ferroptosis, protects hippocampal neurons after ischemia/reperfusion in gerbils by reducing the levels of ROS, iron, and TfR1 protein and increasing the expression levels of GPX4 and Fpn1 protein [92]. (6) Edaravone Dexbomeol is a compound of edaravone and dexcamphenol. It has been demonstrated that this novel neuroprotective drug improves the prognosis of acute IS patients by inhibiting the expression of iROS and TNF-α. In a phase II clinical trial, NCT01929096, its safety and tolerability are better than edaravone used alone [93].

## Inflammatory Responses and Neuronal Death

Inflammatory responses cover the whole process from the acute stage to convalescence in IS. On the one hand, the inflammatory response puts pressure on brain cells and causes neuron death, and on the other hand, contributes to tissue repair as one part of the innate defense system. In the acute phase of IS, the inflammatory response causes irreversible brain damage.

The inflammatory response starts as early as the onset of ischemia. As noted above, oxidative stress and excitotoxicity occur immediately when the blood vessels in the brain are blocked, with the activation of microglia proceeding within minutes. In parallel, damage-associated molecular patterns (DAMPs) released by dead cells, such as purines, also activate microglia [94]. Subsequently, activated microglia release numerous pro-inflammatory factors which recruit peripheral immune cells and activate other brain cells such as astrocytes. Pro-inflammatory factors promote ICAM-1 and selectin expression on endothelial cells, making it easier for peripheral immune cells to reach the ischemic area. Recruited peripheral immune cells also secrete pro-inflammatory factors [95].

Astrocytes also contribute to the inflammatory response and exacerbate damage to neurons after IS. Cytokines released by activated microglia like IL-1α, TNF-α, and C1q induce the activation of reactive astrocytes and the subsequent production of pro-inflammatory mediators including IL-1, TNF-α, IL-6, interferon γ (IFN-γ), MMP-9, radicals, and chemokines like chemokine (C-C motif) ligand 2 [96, 97]. MMP-9 not only damages brain tissue but also disrupts the blood-brain barrier, leading to peripheral immune cell infiltration [98]. ATP released by dead cells activates the inflammasome *via* astrocytic pannexin 1 channels [99]. In addition to pattern recognition receptors, astrocytes also express the class II major histocompatibility complex (MHC II) which is the initiator of the T and B lymphocyte-mediated adaptive immune response. The antigen in reactive astrocytes presented by MHC II is induced by the pro-inflammatory cytokine IFN-γ and associated with increased lysosomal exocytosis [100].

In general, pro-inflammatory factors, glial cells, and immune cells form a positive feedback loop to further exacerbate brain damage.

### Inflammatory Responses and Phagocytosis

In addition to inducing inflammation, microglia are also capable of consuming dead and dying neurons [101]. Neurons that suffer from hypoxia and stress expose phosphatidylserine, which is recognized as an “eat-me” signal by microglia [102]. Phagocytosis seems to be beneficial. However, it has been found that living neurons in the ischemic penumbra are also engulfed by microglia, leading to delayed neuronal death at least 24 h after cerebral ischemia. Blocking this phagocytosis strongly reduces neuronal loss after IS [103]. Phosphatidylserine blockade rescues up to 90% of neurons in primary rat cultures stimulated by lipoteichoic acid or lipopolysaccharide (LPS) [104]. Activated microglia also display sialidase activity, leading to the surface deacetylation of microglia and the complement receptor 3-mediated phagocytosis of neurons [105].

### Inflammatory Responses and Apoptosis by the Extrinsic/Death Receptor Pathway

**Fig. 5:**
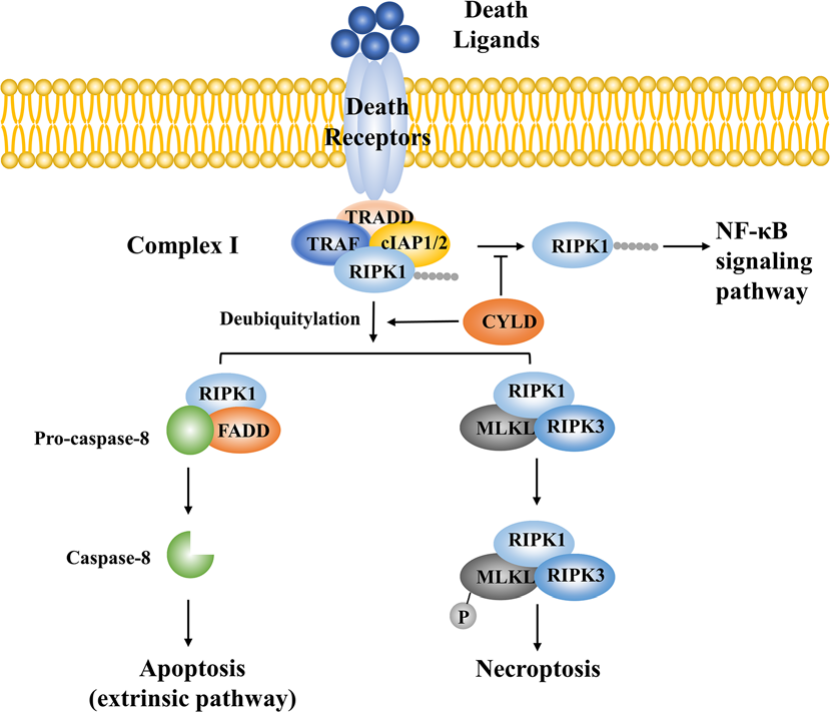
Death ligands induce apoptosis and necroptosis. Death ligands (TNF-α, FASL, and TRAIL) bind to death receptors. CYLD de-ubiquitylates RIPK1 and the rest of complex I recruits FADD. FADD homodimerizes caspase-8, resulting in apoptosis. RIPK1 activation contributes to the formation of the RIPK1–RIPK3–MLKL complex. RIPK3-mediated phosphorylation of MLKL induces necroptosis. Death receptors also triggers NF-κB signaling *via* RIPK1 ubiquitylation by cIAP1/2. CYLD inhibits this process. FADD, Fas-associated death domain; cIAP1/2, cellular inhibitor of apoptosis 1/2; MLKL, mixed lineage kinase domain-like; RIPK, receptor interacting serine/threonine kinases; TRADD, tumor necrosis factor receptor type 1-associated death domain; TRAF, TNFR-associated factor.

Exogenous apoptosis is triggered by TNF-α, FASL and TRAIL. These ligands bind to death receptors with death domains including TNF-R1, Fas, and TRAIL-R. Take TNF-α as an example. The death domain is a critical structure connecting downstream complex I [TRADD (tumor necrosis factor receptor type 1-associated death domain), TRAF2, cIAP1/2, and RIPK1] after ligation of TNF-R1. CYLD de-ubiquitylates RIPK1 and the rest of complex I recruits FADD (Fas-Associated protein with Death Domain) *via* homotypic death domain interactions [106]. FADD recruits and then homodimerizes caspase-8, triggers the caspase cascade, and results in mitochondrial membrane leakage, DNA cleavage, and apoptosis in neuronal cells [107]. TNF-R1 also triggers NF-κB signaling *via* RIPK1 ubiquitylation by cIAP1/2. The limited amount of RIPK1 might explain the crosstalk of NF-κB signaling and the apoptosis pathway emanating from TNF-R1. The sensitivity of cells to TNF-induced apoptosis increases when NF-κB activity is absent, while artificial activation of NF-κB prevents apoptosis (Fig. 5).

### Inflammatory Responses and Necroptosis

Necroptosis is one type of lytic-programmed cell death. In addition to apoptosis, TNF-α also triggers neuronal necroptosis after IS. Necroptosis signal conduction is triggered by death receptors such as TNFR1, TRAILR, Fas, IFNR, and TLR3/4 [108]. The activation of death receptors results in the de-ubiquitylation of receptor-interacting protein kinase 1 (RIPK1) by the de-ubiquitinating enzyme CYLD [109]. After RIPK1 activation, the preserved complex I recruits RIPK3 *via* homotypic RHIM domain interactions, consequently forming the RIPK1-RIPK3-MLKL (mixed lineage kinase domain-like) signaling complex. MLKL forms a homotrimer and subsequently locates to the cell plasma membrane. RIPK3-mediated phosphorylation of MLKL induces necroptosis [110].

Recently, growing numbers of molecules have been discovered to modulate TNF-induced neuronal necroptosis. Jinho *et al.* revealed that CHIP (carboxyl terminus of Hsp70-interacting protein) negatively regulates RIPK3 and RIPK1 *via* E3 ligase-mediated ubiquitylation. CHIP-depleted cells express higher levels of RIPK3 and exhibit enhanced sensitivity to necroptosis induced by TNF-α [111]. Accumulating evidence has demonstrated that CHIP is involved in the pathological progression of IS through different mechanisms [112]. CHIP overexpression prevents neuronal degeneration *in vitro* and *in vivo* [113]. These findings suggest that targeting key molecules in necroptosis signaling may be effective for treating neuronal injury after IS.

### Inflammatory Responses and Pyroptosis

Pyroptosis is a gasdermin D (GSDMD)-mediated form of regulated cell death featuring continuous cell expansion until the cell membrane ruptures, resulting in the release of cell contents and subsequent strong inflammatory responses. Pyroptosis is classified into the caspase-1-dependent classical pathway and caspase-4/5/11-dependent pathways. Pathogen-associated pattern molecules and DAMPs are recognized by pattern recognition receptors, which subsequently assemble into inflammasomes that recruit caspase-1 for self-splicing. Activated caspase-1 cleaves GSDMD to form the GSDMD nitrogen terminus and the carbon terminus, which binds to the phospholipid proteins on the cell membrane to form pores, release the contents, and induce pyroptosis. Activated caspase-1 also cleaves pro-IL-1β and pro-IL-18 to form active IL-1β and IL-18, and releases them into the extracellular space, causing an inflammatory response. Intracellular caspase-4/5/11 directly binds to LPS to undergo auto-oligomeric activation. They cleave GSDMD and cause pyroptosis [114].

**Fig. 6:**
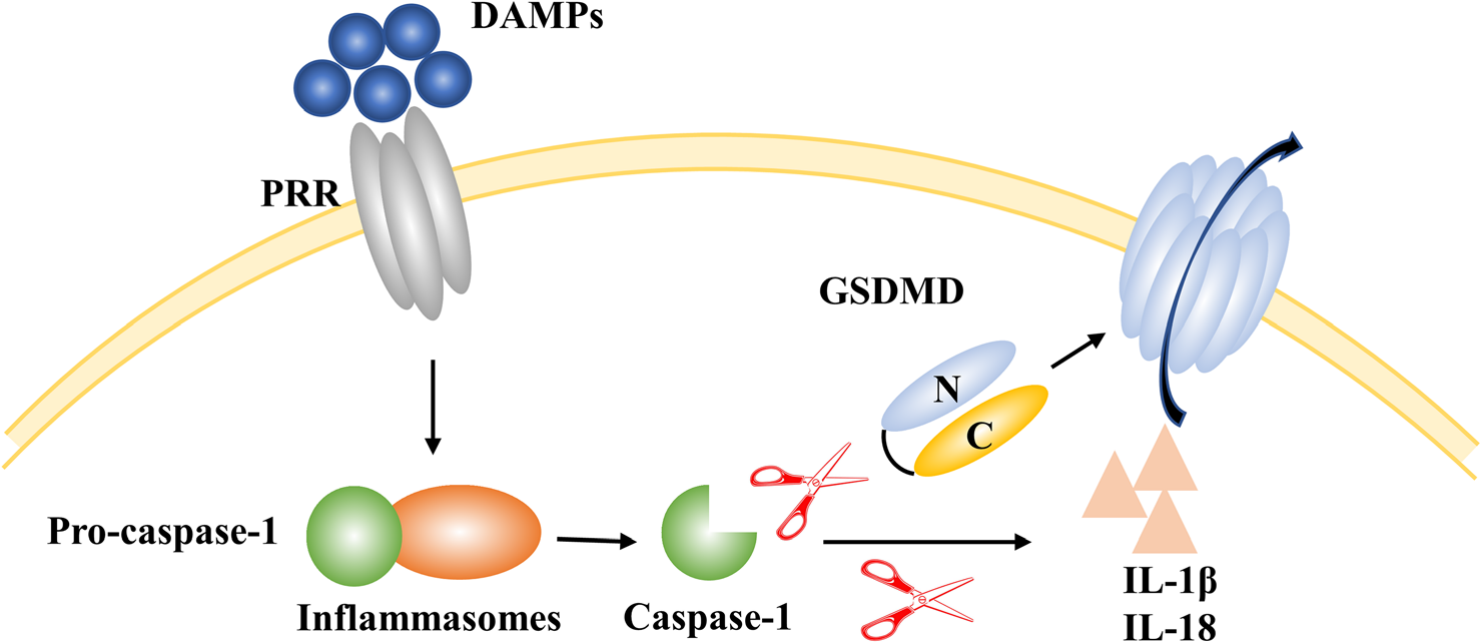
Pyroptosis during ischemic stroke. DAMPs bind to PRRs, resulting in the formation of inflammasomes and the production of caspase-1. On the one hand, caspase-1 cleaves GSDMD to form pores on the cell membrane, resulting in content release and pyroptosis. On the other hand, caspase-1 cleaves pro-IL-1β and pro-IL-18 to form active IL-1β and IL-18, causing inflammatory responses. GSDMD, gasdermin D; DAMPs, damage-associated molecular patterns; PRR, pattern recognition receptor.

Pyroptosis actively participates in the inflammatory response and contributes to neuronal death during IS. An elevated level of pyroptosis has been reported around the infarcted area in the early stage of ischemia-reperfusion. The ablation of GSDMD significantly reduces pyroptosis and the infraction volume after ischemia/reperfusion by inhibiting the secretion of mature IL-18 and IL-1β from microglia [115]. Wang *et al.* revealed that melatonin-treated exosomes effectively reduce the activation of caspase-1 and inflammasome-mediated neuronal pyroptosis through the TLR4/NF-κB signaling pathway, therefore decreasing the infarct volume and improving recovery from the functional deficit [116]. Lammert *et al.* reported that pyroptosis induced by the AIM2 inflammasome results in the elimination of genetically compromised neuronal cells during neurodevelopment [117] (Fig. 6).

### Therapeutics of Inflammatory Responses in Ischemic Stroke

#### Targeting Microglia

Emerging evidence points out that target microglia are effective in alleviating the inflammatory response and neuronal injury after IS. Zhang *et al.* reported that miR-146a-5p attenuates microglia-mediated neuroinflammation, leading to a reduction in infarct volume and neural deficits through the IRAK1/TRAF6 pathway [118]. Gentianine, an alkaloid obtained from *Gentiana scabra* Bunge, inhibits the activation of microglia and ameliorates inflammatory responses *via* the TLR4/NF-κB signaling pathway in a transient MCAO model [119]. Growing numbers of studies have shown that lncRNAs regulate the inflammation and polarization of microglia during cerebral ischemia-reperfusion injury [120]. *In vitro*, SNHG4 up-regulates STAT6 and represses inflammation by adsorbing miR-449c-5p in microglia [121]. Knockdown of the LncRNA MEG3 inhibits M1 polarization and inflammation and promotes M2 polarization through Krüppel-like factor 4 *in vitro* and *in vivo* [122]. Jin *et al.* revealed that lncRNA NEAT1 is strongly correlated with activated-microglia mRNAs *via* the transcriptome-wide analysis of mice with focal ischemia. Knockdown of Neat1 significantly represses the activation of microglia and subsequent brain damage caused by pro-inflammatory cytokines released by microglia [123]. Knockdown of the lncRNA NEAT1 also alleviates the apoptosis of N2a cells and increases neuronal viability [124].

#### Targeting Immune Cells

Several genes in immune cells have been shown to play crucial roles in modulating the inflammatory response in the ischemic brain. Genetic deletion of PKM2 in myeloid cells reduces peripheral neutrophil infiltration and inflammatory cytokines following cerebral ischemia-reperfusion. Inhibition of the nuclear translocation of PKM2 significantly reduces neutrophil hyperactivation and improves functional deficit recovery following stroke [125]. Meng *et al.* reported that infiltrating CD3^+^CD4^−^CD8^−^ T cells enhance immune and inflammatory responses, resulting in destructive ischemic brain injury *via* modulating the FasL/PTPN2/TNF-α signaling pathway [126]. Weitbrecht revealed that CD4^+^ T cells facilitate delayed B cell infiltration into the brain and CD4 depletion attenuates post-stroke cognitive impairment [127]. Nakajima *et al.* reported that mucosa-associated invariant T cell deficiency suppresses the activation of microglia and attenuates cytokine production, including IL‐1β and IL‐6 in a transient MCAO model [128]. Small extracellular vesicles derived from embryonic stem cells trigger the TGF-β/Smad pathway in CD4^+^ T cells, and thereby decrease the inflammatory response and neuronal death after IS [129].

#### Targeting Pro-inflammatory Mediators

Growing evidence suggests that therapy against pro-inflammatory mediators is potent in alleviating inflammation during IS. The confinement of MMP-9 expression *via* lentivirus injection of hypoxia response element on day 7 after transient MCAO contributes to improved neurological outcomes, increased peri-infarct microvessels, and alleviation of ischemia-induced brain atrophy [130]. Nanoparticles of the pharmacologically active oligosaccharide material TPCD reduces the expression of pro-inflammatory cytokines (TNF-α, IL-1β, and IL-6) and inhibits neuronal apoptosis [131]. The TNF-α receptor inhibitor R-7050 reverses the activation of TNF receptor-I, NF-κB, and IL-6, and reduces the metabolic alterations in a rat model of permanent cerebral ischemia [132]. Silencing of lncRNA SNHG15 decreases the levels of pro-inflammatory cytokines (TNF-α and IL-1β) and apoptosis of N2a cells *via* sequestering miR-18a and subsequently activating the ERK signaling pathway [133]. Several immunomodulatory drugs are in clinical trials. A recent randomized controlled phase II trial has shown that an IL-1 receptor antagonist reduced inflammation and improved the clinical outcome in 80 patients within 5 h of IS onset, indicating the potential clinical applications of targeted inflammatory drugs [134].

## Autophagy and Autophagy-Dependent Cell Death (ADCD)

Autophagy, a term first coined by Christian de Duve in the 1960s [135], refers to a conserved intracellular catabolic degradation pathway that is activated in response to starvation and stress [136]. By delivering cytoplasmic constituents to lysosomes and digesting them, cells can maintain homeostasis by recycling degraded metabolic elements. The process is vital to the survival of cells [137, 138]. However, it is well-known that a dual effect is manifested in autophagy. In detail, excessive activation of autophagy has a detrimental impact on cellular functions and then causes cell injury or even death [139]. Similarly, the role of autophagy underlying IS remains unclear since it serves as a double-edged sword that can either protect or damage neurons upon ischemic insult [140]. In fact, either impaired or excessive induction of autophagy leads to neuronal cell injury, and the boundary between lethal and non-lethal basal autophagy remains vague.

Recently, the concept of ADCD has been put forward and sparked a heated debate. The Nomenclature Committee of Cell Death defines ADCD as ‘a form of regulated cell death that mechanistically depends on the autophagic machinery (or components thereof)’ [141]. This concept describes cell death exclusively caused by autophagy, without the involvement of other death mechanisms like apoptosis or necroptosis. However, the concept is severely challenged given the widespread interplay between autophagy and apoptosis or necrosis [142]. Moreover, historically, morphological criteria are solely applied to distinguish ‘autophagic cell death’, but they have been confirmed to be misleading [143, 144]. According to the Nomenclature Committee, only when at least two molecular components associated with autophagy are genetically silenced to block cell death is this phenomenon defined as ADCD. The most well-accepted form of ADCD is ‘autosis’, which was named by Levine and his colleagues in 2015 and is dependent on Na^+^/K^+^-ATPase [145].

### Role of Autophagy in Ischemic Stroke

In effect, the role of autophagy in IS has never been clearly defined and described: some studies have shown autophagy to be deleterious in IS while others consider it to be neuroprotective. Here, we review some of these findings and focus on the negative effects of autophagy [146].

The neuronal deletion of Atg7 in mice prevents hypoxia-induced neuronal autophagy and reduces neuronal death in multiple brain regions, and thereby results in a 42% decrease of tissue loss compared to wild-type mice. Increased numbers of microtubule-associated protein 1 light chain 3-, lysosomal-associated membrane protein 1-, and cathepsin D-positive cells have also been found, indicating upregulated autophagy after severe ischemic insult [147]. Overexpression of TP53-induced glycolysis and apoptosis regulator (TIGAR) significantly reduces the activation of ischemia/reperfusion-induced autophagy, alleviates brain damage, and protects against neuronal injury by suppressing autophagy through upregulating phosphorylated mTOR and S6KP70 [148]. Moreover, dysfunctional lysosomal storage is associated with the early burst of autophagy, then inducing synaptic impairment in neurons [149]. Besides, autophagy is considered to be involved in blood-brain barrier disruption [150]. Enhanced autophagic activity and loss of occludin have been reported in brain endothelial cells in a mouse model of MCAO; this ultimately resulted in permeability changes and contributed to an amplification of ischemic brain damage [150]. The activation of autophagy can also activate the cathepsin-tBid-mitochondrial apoptotic signaling pathway by losing stabilization of the lysosomal membrane in ischemic cells [151].

Autophagy may induce apoptosis and eventually result in neuronal cell death through these pathways: Primarily, p53 upregulates transactivating damage-regulated autophagy modulator 1 (DRAM), triggers mitochondrial outer membrane permeabilization and upregulates pro-apoptotic genes such as Bax and Bak [152, 153]. In addition, BH3-only proteins and BH3 mimics competitively bind to Bcl-2 and induce autophagy. This then elicits the inhibition of anti-apoptotic proteins like Bcl-2 and Bcl-X_L_ and activation of pro-apoptotic members such as Bax and Bak [154, 155]. Another category is Ser/Thr kinases which includes DAPK, JNK, and protein kinase B (PKB/AKT). Among them, DAPK and AKT play similar roles as BH3-only proteins. Activation of p19ARF along with p53 and protein phosphatase 2A is also involved in DAPK pathways [155]. Besides, oncogenes like MYC and RAS, as well as ROS, ceramide, and Ca^2+^ overload are somewhat considered to regulate both autophagy and apoptosis [155, 156].

### Induction of Autophagy in Ischemic Stroke

As discussed above, a variety of stress factors occur in IS. Pathological changes like excitotoxicity, oxidative stress, and inflammatory responses all trigger multiple signaling cascades of autophagy. Then, excessive initiation of autophagy and subsequent cellular death occur on account of these stimulants [146, 157].

**Fig. 7:**
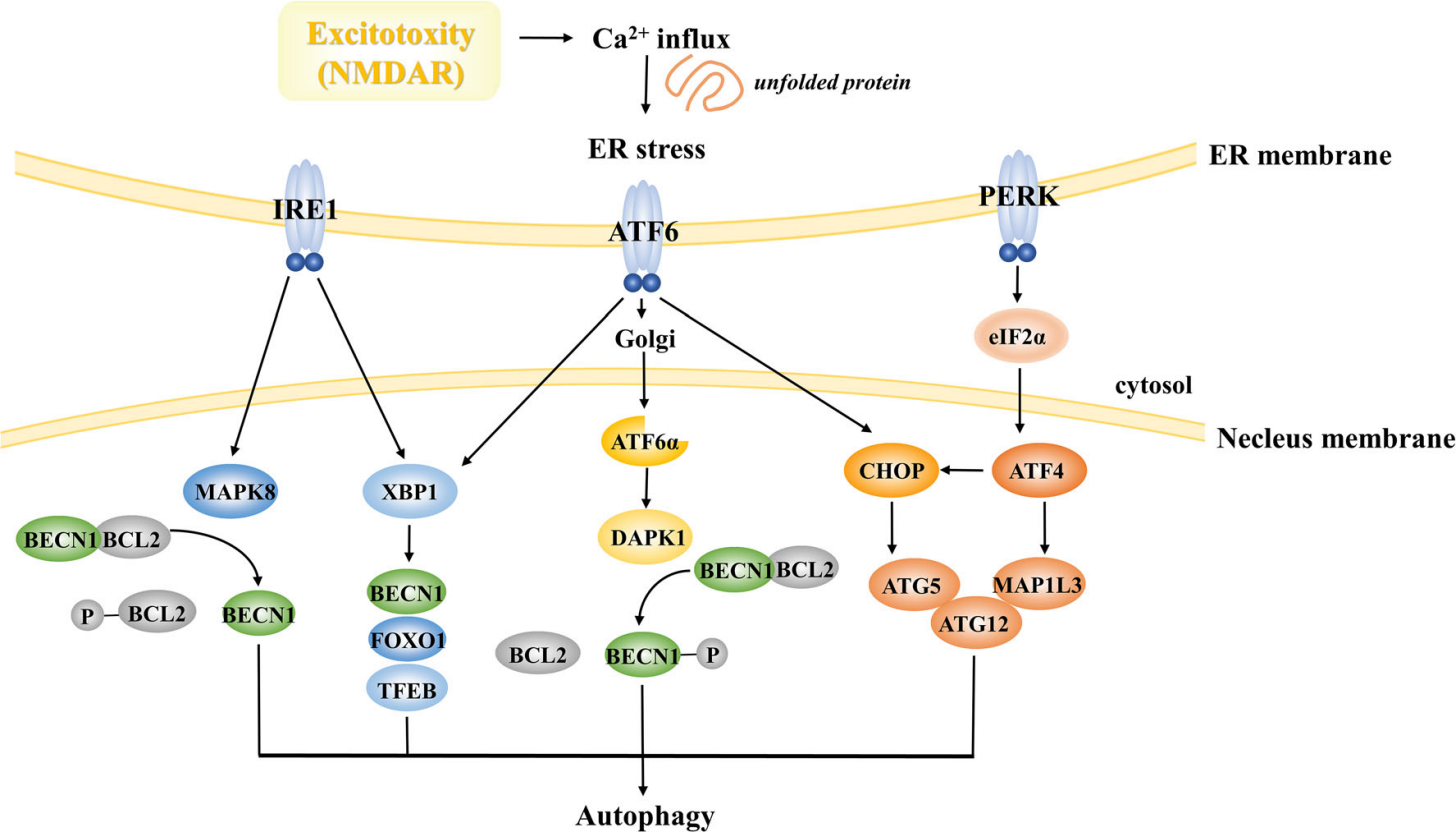
Autophagy induced by excitotoxicity. NMDAR leads to Ca^2+^ influx, causing unfolded protein and ER stress. The three transmembrane sensors IRE1, ATF6, and PERK activate a complex cascade with autophagic induction. IRE1 upregulates BECN1 *via* MAPK8 and XBP1. XBP1 also transactivates FOXO1 and TFEB, bypassing BECN1, and induces autophagy. PERK upregulates ATF4 and CHOP *via* the eIF2α axis, then enhances the transcription of MAP1LC3, ATG5, and ATG12. ATF6 upregulates the DAPK1-mediated phosphorylation of BECN1 and is believed to activate both XBP1 and CHOP. IRE1, inositol-requiring enzyme 1; ATF, activating transcription factor; PERK, PKR-like endoplasmic reticulum kinase; MAPK8, mitogen-activated protein kinase 8; XBP1, box-binding protein 1; FOXO1, forkhead box O1; TFEB, transcription factor EB; CHOP, CCAAT/enhancer binding protein homologous protein; eIF2α, eukaryotic initiation factor 2α; MAP1LC3, microtubule associated protein 1 light chain 3 beta; DAPK1, death-associated protein kinase 1.

Overactivated NMDARs can bring about increased intracellular Ca^2+^ as well as redox imbalance [3]. Such alterations are able to cause unfolded or misfolded proteins to accumulate in the endoplasmic reticulum (ER) lumen, leading to ER stress [158]. Afterwards, maladaptive autophagy is initiated *via* three major transmembrane sensors, among which, PERK and its downstream mediators like ATF4 and DDIT3 promote autophagosome formation by upregulating the transcription of microtubule associated protein 1 light chain 3 beta (MAP1LC3) and ATG5 [159]. IRE1 has been reported to cause upregulation of BECN1 through X box-binding protein 1 (XBP1) and mitogen-activated protein kinase 8 (MAPK8) [160]. This leads to the induction of excessive autophagy. Furthermore, ATF6 participates in autophagic initiation through upregulating the DAPK1-mediated phosphorylation of BECN1 [160, 161]. It also has an effect on the activation of CCAAT/enhancer binding protein homologous protein (CHOP) and XBP1, which are able to stimulate overactive autophagy [160] (Fig. 7).

**Fig. 8:**
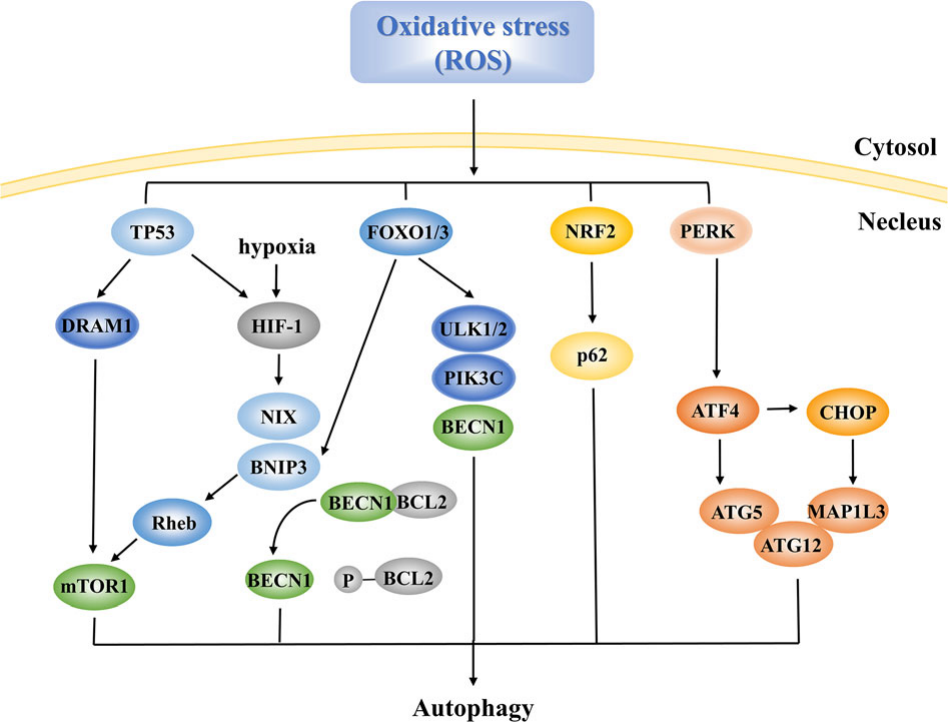
Autophagy induced by oxidative stress. Increased ROS upregulates nuclear p53, transactivates DRAM1, inhibits mTOR1, and activates HIF-1. Besides, HIF-1 due to oxygen deprivation triggers the transcription of BNIP3 and NIX. BNIP3 also inhibits mTOR1 *via* inhibiting Rheb. The FOXO family, especially FOXO1 and FOXO3, upregulates ATGs including ULK1/2, BECN1, and PIK3C, as well as BNIP3. ROS also upregulates NRF2 to enhance the levels of p62 and upregulates PERK as shown in Fig. 6. DRAM1, damage-regulated autophagy modulator1; mTOR1, mechanistic target of rapamycin complex 1; HIF-1, hypoxia inducible factor 1; BNIP3, BCL2/adenovirus E1B 19 kDa interacting protein 3; NIX, BNIP3-like; Rheb, Ras homolog protein enriched in brain; FOXO, forkhead box O; ULK1/2, Unc-51 like autophagy activating kinase; NRF2, NF-E2-related factor 2.

Oxidative stress is triggered by accumulation of ROS. Increased ROS upregulates nuclear p53, and then facilitates autophagy by transactivating damage-regulated autophagy modulator1, inhibiting mechanistic target of rapamycin complex 1, and activating hypoxia inducible factor1 (HIF-1) [162, 163]. In addition, activation of HIF-1 triggers autophagy by enhancing the transcription of BCL2/adenovirus E1B 19 kDa interacting protein 3 (BNIP3) and BNIP3-like (BNIP3L/NIX) [164]. BNIP3 also induces autophagy by directly inhibiting Ras homolog protein enriched in brain [165]. Moreover, the forkhead box O (FOXO) family of transcription factors, especially FOXO1 and FOXO3, play a role in autophagic induction by upregulating the expression of various ATGs including ULK1/2, BECN1, and PIK3C as well as BNIP3 [164, 166]. Besides, ROS leads to the upregulation of NF-E2-related factor 2, which enhances the expression of p62 and induces excessive autophagy [167]. Furthermore, ROS accumulation induces the transcription of PERK, whose effects on autophagy are described in detail above [168] (Fig. 8).

It is well accepted that autophagy plays a positive role in suppressing the inflammatory response. However, autophagy can also upregulate inflammation in an unconventional way [169]. And in turn, inflammasomes in neuronal cells can trigger the formation of autophagosomes and activate autophagy [170]. Since exogenous apoptosis is triggered by inflammation, autophagy is induced subsequently through apoptosis-dependent pathways like p53, BH3-only proteins, DAPK, JNK, and PKB. Besides, persistent or excessive activation of the inflammatory response leads to the pathological stimulation of autophagy [171].

### Therapeutics of Autophagy in Ischemic Stroke

#### Targeting Excessive Autophagy

As noted above, neuronal deletion of Atg7, as well as TIGAR, schizandrin, and dexmedetomidine (DEX) is thought to be neuroprotective in IS *via* suppressing autophagy. Apart from these, the most acknowledged inhibitor is 3-methyladenine, which inhibits PI3K. However, it only experimental now and its effect *in vivo* remains doubtful [172]. The ability of ULK1/2 inhibitors such as SBI-0206965 and ULK101 to down-regulate autophagy is promising [172, 173]. Moreover, chloroquine and hydroxychloroquine, which have been widely demonstrated to be safe in cancer therapy, are considered to act on the inhibition of autophagy [174]. Propofol at a dose of 100 μmol/L is reported to significantly reduce BECN1 and ULK1, as well as inhibit the Ca^2+^/CaMKKβ/AMPK/mTOR axis [175, 176]. Besides, VSP34 inhibitors include SAR405, compound 13, and SB02024. Among them, SB02024 is highly potent and is a promising candidate for clinical therapeutics. In addition, DEX is thought to inhibit autophagy in IS by upregulating CCND1, SQSTM1, and HIF1A, and downregulating LC3A, BECN1, BAX, and DRAM [176]. Intriguingly, lithium, initially a drug for bipolar disorder, has been reported to suppress autophagy by inhibiting PI3K and upregulating Bcl-2 [177]. Melatonin is also thought to effectively inhibit autophagy *via* modulating a decrease in ER stress [14].

#### Targeting Adaptive Autophagy

As the first drug identified in autophagy induction, rapamycin has been widely examined in the context of IS, along with its analogues such as temsirolimus. Unfortunately, it has multiple adverse effects, given the autophagy-independent functions of mTOR [14, 178]. In addition, metformin, a drug used for diabetes, has been reported to activate AMPK through mitochondrial depletion of ATP. Aspirin also induces autophagy *via* upregulating transcription factor EB in brain cells [179]. Other compounds like rilmenidine, eugenol, progesterone, and resveratrol have been reported to induce adaptive autophagy in IS [176]. Besides, knockdown of circular RNA Hectd1 expression significantly decreases the infarct area, attenuates neuronal deficits, and ameliorates astrocyte activation by activating autophagy in transient MCAO mice [180]. Moreover, overexpression of poly (ADP-ribose) polymerase family, member 14 (PARP14) alleviates microglial activation by modulating microglial autophagy and thus promotes post-stroke functional recovery [181].

## Conclusions and Future Directions

In this review, we summarize the pathological role of excitotoxicity, oxidative stress, and inflammatory responses and the involvement of multiple death modes mediated by these deleterious events in IS. Despite all of the data, the exact sequence of these pathological events has not yet been completely clarified according to current studies. It should also be pointed out that this review does not cover some forms of neuronal death that are rarely found and that could not be directly summarized above, such as paraptosis.

The deep understanding of the intricate mechanisms based on research over the past decades provides latent treatment options. We point out some possible future directions, or problems that need to be solved urgently.

Future studies can pay more attention to combining NMDAR-targeted agents with stem cell therapy. Stem cell therapy following ischemic insults has proved to be a novel and promising therapeutic strategy in recent years. It promotes CNS plasticity and regeneration, angiogenesis, and immunomodulation [182]. The efficacy and safety of cell-based therapy have been confirmed in animal models. However, evidence of its neuroprotective effect is yet insufficient in humans. Several drugs that are able to enhance neurogenesis may assist stem cell therapy, such as above-mentioned Tat-NR2B-9c [183].

Oxidative stress causes damage to neurons during the occurrence of IS and in subsequent treatment, blood reperfusion also produces large amounts of ROS leading to neuronal damage. Therefore, how to reduce ROS generation or how to block the ROS cascade will be closely related to the prognosis.

It is worth noting that the inflammatory response is a double-edged sword. Microglia are able to release pro-inflammatory or anti-inflammatory cytokines at different times and in distinct states, resulting in detrimental or beneficial effects [184]. Proliferating astrocytes also form a physical barrier against inflammation *via* upregulating intermediate filament proteins and secreting extracellular matrix components [97]. The physical barrier limits inflammation in the early stage of ischemia. However, it has been shown that the glial scar around the ischemic region prevents axonal outgrowth in the late period. Astrocytes also contribute to long-term recovery after IS by secreting neurotrophic factors [185]. How to modulate the participation of astrocytes in the inflammatory response to minimize neuronal injury is a challenging question. Hence, where and when to intervene in the inflammatory process remains to be studied.

Multiple therapeutics against autophagy have been reported recently. However, given the presence of both pro-survival and pro-death autophagy in IS, the management targeting proper sites needs to be demonstrated. Some studies have shown that in the early stage of ischemia, well-functioning basal autophagy dominates, and that lethal autophagy develops with prolonged initiation, especially after reperfusion [186]. But the cut-off point remains vague and further exploration is required due to the paucity of available evidence. Besides, inhibition of one specific regulatory molecule is never sufficient to suppress the whole process of autophagy. This fact therefore lowers the reliability of many current studies. Another puzzle involved in autophagy then emerges: intricate cross-links between autophagy and other mechanisms. This complexity makes it challenging to regulate autophagy properly, and enormous blanks in ADCD remain to be explored

In addition, future investigations may focus on the exploration of multi-target brain protectants and the feasibility of their clinical application.

## References

[1] Gorelick PB (2019). The global burden of stroke: Persistent and disabling. Lancet Neurol.

[2] Fricker M, Tolkovsky AM, Borutaite V, Coleman M, Brown GC (2018). Neuronal cell death. Physiol Rev.

[3] Ge Y, Chen WL, Axerio-Cilies P, Wang YT (2020). NMDARs in cell survival and death: Implications in stroke pathogenesis and treatment. Trends Mol Med.

[4] Lau A, Tymianski M (2010). Glutamate receptors, neurotoxicity and neurodegeneration. Pflugers Arch.

[5] Wu QJ, Tymianski M (2018). Targeting NMDA receptors in stroke: New hope in neuroprotection. Mol Brain.

[6] Lai TW, Zhang S, Wang YT (2014). Excitotoxicity and stroke: Identifying novel targets for neuroprotection. Prog Neurobiol.

[7] Zhou XJ, Chen ZY, Yun WW, Wang HB (2015). NMDA receptor activity determines neuronal fate: Location or number?. Rev Neurosci.

[8] Biegon A, Liraz-Zaltsman S, Shohami E (2018). Stimulation of N-methyl-D-aspartate receptors by exogenous and endogenous ligands improves outcome of brain injury. Curr Opin Neurol.

[9] Kerr JF, Wyllie AH, Currie AR (1972). Apoptosis: a basic biological phenomenon with wide-ranging implications in tissue kinetics. Br J Cancer.

[10] Cheng YR, Jiang BY, Chen CC (2018). Acid-sensing ion channels: Dual function proteins for chemo-sensing and mechano-sensing. J Biomed Sci.

[11] Liu L, Gu LJ, Chen ML, Zheng YY, Xiong XX, Zhu SM (2020). Novel targets for stroke therapy: Special focus on TRPC channels and TRPC6. Front Aging Neurosci.

[12] Piccirillo S, Magi S, Castaldo P, Preziuso A, Lariccia V, Amoroso S (2020). NCX and EAAT transporters in ischemia: At the crossroad between glutamate metabolism and cell survival. Cell Calcium.

[13] Curcio M, Salazar IL, Mele M, Canzoniero LMT, Duarte CB (2016). Calpains and neuronal damage in the ischemic brain: The Swiss knife in synaptic injury. Prog Neurobiol.

[14] Tuo QZ, Zhang ST, Lei P (2022). Mechanisms of neuronal cell death in ischemic stroke and their therapeutic implications. Med Res Rev.

[15] Salvador-Gallego R, Mund M, Cosentino K, Schneider J, Unsay J, Schraermeyer U (2016). Bax assembly into rings and arcs in apoptotic mitochondria is linked to membrane pores. EMBO J.

[16] Dorstyn L, Akey CW, Kumar S (2018). New insights into apoptosome structure and function. Cell Death Differ.

[17] Shakeri R, Kheirollahi A, Davoodi J (2017). Apaf-1: Regulation and function in cell death. Biochimie.

[18] Wang S, Shi XD, Li H, Pang P, Pei L, Shen HY (2017). DAPK_1_ signaling pathways in stroke: From mechanisms to therapies. Mol Neurobiol.

[19] Parsons R. Discovery of the PTEN tumor suppressor and its connection to the PI3K and AKT oncogenes. Cold Spring Harb Perspect Med 2020, 10: a036129. 10.1101/cshperspect.a036129 PMC7397838 31932465

[20] Zhang S, Taghibiglou C, Girling K, Dong ZF, Lin SZ, Lee W (2013). Critical role of increased PTEN nuclear translocation in excitotoxic and ischemic neuronal injuries. J Neurosci.

[21] Shvedova M, Anfinogenova Y, Atochina-Vasserman EN, Schepetkin IA, Atochin DN (2018). C-Jun N-terminal kinases (JNKs) in myocardial and cerebral ischemia/reperfusion injury. Front Pharmacol.

[22] Dixon SJ, Lemberg KM, Lamprecht MR, Skouta R, Zaitsev EM, Gleason CE (2012). Ferroptosis: an iron-dependent form of nonapoptotic cell death. Cell.

[23] Yang WS, Kim KJ, Gaschler MM, Patel M, Shchepinov MS, Stockwell BR (2016). Peroxidation of polyunsaturated fatty acids by lipoxygenases drives ferroptosis. Proc Natl Acad Sci U S A.

[24] Degregorio-Rocasolano N, Martí-Sistac O, Gasull T (2019). Deciphering the iron side of stroke: Neurodegeneration at the crossroads between iron dyshomeostasis, excitotoxicity, and ferroptosis. Front Neurosci.

[25] Cheah JH, Kim SF, Hester LD, Clancy KW, Patterson SE, Papadopoulos V (2006). NMDA receptor-nitric oxide transmission mediates neuronal iron homeostasis *via* the GTPase Dexras1. Neuron.

[26] Jin YZ, Zhuang YX, Liu M, Che JX, Dong XW (2021). Inhibiting ferroptosis: A novel approach for stroke therapeutics. Drug Discov Today.

[27] Li J, Cao F, Yin HL, Huang ZJ, Lin ZT, Mao N (2020). Ferroptosis: past, present and future. Cell Death Dis.

[28] Kita Y, Shindou H, Shimizu T (2019). Cytosolic phospholipase A_2_ and lysophospholipid acyltransferases. Biochim Biophys Acta Mol Cell Biol Lipids.

[29] Ren JX, Sun X, Yan XL, Guo ZN, Yang Y (2020). Ferroptosis in neurological diseases. Front Cell Neurosci.

[30] Pazzaglia S, Pioli C (2019). Multifaceted role of PARP-1 in DNA repair and inflammation: Pathological and therapeutic implications in cancer and non-cancer diseases. Cells.

[31] Andrabi SA, Dawson TM, Dawson VL (2008). Mitochondrial and nuclear cross talk in cell death: Parthanatos. Ann N Y Acad Sci.

[32] Wang XZ, Ge PF (2020). Parthanatos in the pathogenesis of nervous system diseases. Neuroscience.

[33] Kornau HC, Schenker LT, Kennedy MB, Seeburg PH (1995). Domain interaction between NMDA receptor subunits and the postsynaptic density protein PSD-95. Science.

[34] Murciano-Calles J, Coello A, Cámara-Artigas A, Martinez JC (2020). PDZ/PDZ interaction between PSD-95 and nNOS neuronal proteins: A thermodynamic analysis of the PSD95-PDZ2/nNOS-PDZ interaction. J Mol Recognit.

[35] Rong R, Yang H, Rong LQ, Wei XE, Li QJ, Liu XM (2016). Proteomic analysis of PSD-93 knockout mice following the induction of ischemic cerebral injury. Neurotoxicology.

[36] Alano CC, Garnier P, Ying WH, Higashi Y, Kauppinen TM, Swanson RA (2010). NAD+ depletion is necessary and sufficient for poly(ADP-ribose) polymerase-1-mediated neuronal death. J Neurosci.

[37] Andrabi SA, Kim NS, Yu SW, Wang HM, Koh DW, Sasaki M (2006). Poly(ADP-ribose) (PAR) polymer is a death signal. Proc Natl Acad Sci U S A.

[38] Narne P, Pandey V, Simhadri PK, Phanithi PB (2017). Poly(ADP-ribose)polymerase-1 hyperactivation in neurodegenerative diseases: The death knell tolls for neurons. Semin Cell Dev Biol.

[39] Wang YF, An R, Umanah GK, Park H, Nambiar K, Eacker SM, *et al*. A nuclease that mediates cell death induced by DNA damage and poly(ADP-ribose) polymerase-1. Science 2016, 354: aad6872. 10.1126/science.aad6872 PMC5134926 27846469

[40] Fouquerel E, Goellner EM, Yu ZX, Gagné JP, Barbi de Moura M, Feinstein T, *et al*. ARTD1/PARP1 negatively regulates glycolysis by inhibiting hexokinase 1 independent of NAD+ depletion. Cell Rep 2014, 8: 1819–1831. 10.1016/j.celrep.2014.08.036 PMC4177344 25220464

[41] Yang JH, Vitery M, Chen JN, Osei-Owusu J, Chu JC, Qiu ZZ (2019). Glutamate-releasing SWELL1 channel in astrocytes modulates synaptic transmission and promotes brain damage in stroke. Neuron.

[42] Yin AQ, Guo H, Tao L, Cai GH, Wang YZ, Yao LB (2020). NDRG2 protects the brain from excitotoxicity by facilitating interstitial glutamate uptake. Transl Stroke Res.

[43] Chamorro Á, Dirnagl U, Urra X, Planas AM (2016). Neuroprotection in acute stroke: Targeting excitotoxicity, oxidative and nitrosative stress, and inflammation. Lancet Neurol.

[44] Godino M, Romera VG, Sánchez-Tomero JA, Pacheco J, Canals S, Lerma J (2013). Amelioration of ischemic brain damage by peritoneal dialysis. J Clin Invest.

[45] Park CK, Nehls DG, Graham DI, Teasdale GM, McCulloch J (1988). The glutamate antagonist MK-801 reduces focal ischemic brain damage in the rat. Ann Neurol.

[46] Muir KW (2006). Glutamate-based therapeutic approaches: Clinical trials with NMDA antagonists. Curr Opin Pharmacol.

[47] Fischer G, Mutel V, Trube G, Malherbe P, Kew JN, Mohacsi E (1997). Ro 25–6981, a highly potent and selective blocker of N-methyl-D-aspartate receptors containing the NR2B subunit. Characterization *in vitro*. J Pharmacol Exp Ther.

[48] Chenard BL, Bordner J, Butler TW, Chambers LK, Collins MA, de Costa DL (1995). (1S, 2S)-1-(4-hydroxyphenyl)-2-(4-hydroxy-4-phenylpiperidino)-1-propanol: A potent new neuroprotectant which blocks N-methyl-D-aspartate responses. J Med Chem.

[49] Regan MC, Zhu ZJ, Yuan HJ, Myers SJ, Menaldino DS, Tahirovic YA (2019). Structural elements of a pH-sensitive inhibitor binding site in NMDA receptors. Nat Commun.

[50] Xu QL, Hu MQ, Li JM, Ma XD, Chu ZX, Zhu QH (2022). Discovery of novel brain-penetrant GluN2B NMDAR antagonists via pharmacophore-merging strategy as anti-stroke therapeutic agents. Eur J Med Chem.

[51] Hackos DH, Lupardus PJ, Grand T, Chen YL, Wang TM, Reynen P (2016). Positive allosteric modulators of GluN2A-containing NMDARs with distinct modes of action and impacts on circuit function. Neuron.

[52] Yao LL, Zhou Q (2017). Enhancing NMDA receptor function: Recent progress on allosteric modulators. Neural Plast.

[53] Martinez-Coria H, Arrieta-Cruz I, Cruz ME, López-Valdés HE (2021). Physiopathology of ischemic stroke and its modulation using memantine: Evidence from preclinical stroke. Neural Regen Res.

[54] Seyedsaadat SM, F Kallmes D. Memantine for the treatment of ischemic stroke: Experimental benefits and clinical lack of studies. Rev Neurosci 2019, 30: 203–220. 10.1515/revneuro-2018-0025 30067513

[55] Zhang C, Liu XD, Xu H, Hu GY, Zhang X, Xie Z (2020). Protopanaxadiol ginsenoside Rd protects against NMDA receptor-mediated excitotoxicity by attenuating calcineurin-regulated DAPK_1_ activity. Sci Rep.

[56] Wang S, Chen K, Yu J, Wang XJ, Li Q, Lv F (2020). Presynaptic Caytaxin prevents apoptosis *via* deactivating DAPK_1_ in the acute phase of cerebral ischemic stroke. Exp Neurol.

[57] Tu WH, Xu X, Peng LS, Zhong XF, Zhang WF, Soundarapandian MM (2010). DAPK_1_ interaction with NMDA receptor NR2B subunits mediates brain damage in stroke. Cell.

[58] Pulido R (2018). PTEN inhibition in human disease therapy. Molecules.

[59] Zheng TT, Shi Y, Zhang J, Peng J, Zhang X, Chen KK (2019). miR-130a exerts neuroprotective effects against ischemic stroke through PTEN/PI3K/AKT pathway. Biomed Pharmacother.

[60] Mu JW, Cheng X, Zhong SS, Chen XH, Zhao CS (2020). Neuroprotective effects of miR-532-5p against ischemic stroke. Metab Brain Dis.

[61] Li LJ, Cui PH, Ge HM, Shi YJ, Wu XG, Zhang FR (2020). miR-188-5p inhibits apoptosis of neuronal cells during oxygen-glucose deprivation (OGD)-induced stroke by suppressing PTEN. Exp Mol Pathol.

[62] Yi ZQ, Shi YY, Zhao PW, Xu Y, Pan PL (2020). Overexpression of miR-217-5p protects against oxygen-glucose deprivation/reperfusion-induced neuronal injury *via* inhibition of PTEN. Hum Cell.

[63] Zheng JJ, Dai QX, Han KY, Hong WD, Jia DY, Mo YC (2020). JNK-IN-8, a c-Jun N-terminal kinase inhibitor, improves functional recovery through suppressing neuroinflammation in ischemic stroke. J Cell Physiol.

[64] Xu BT, Xu JP, Cai NB, Li MF, Liu L, Qin YY (2021). Roflumilast prevents ischemic stroke-induced neuronal damage by restricting GSK3β-mediated oxidative stress and IRE1α/TRAF2/JNK pathway. Free Radic Biol Med.

[65] Ballarin B, Tymianski M (2018). Discovery and development of NA-1 for the treatment of acute ischemic stroke. Acta Pharmacol Sin.

[66] Hill MD, Goyal M, Menon BK, Nogueira RG, McTaggart RA, Demchuk AM (2020). Efficacy and safety of nerinetide for the treatment of acute ischaemic stroke (ESCAPE-NA1): A multicentre, double-blind, randomised controlled trial. Lancet.

[67] Ayuso-Dolado S, Esteban-Ortega GM, Vidaurre ÓG, Díaz-Guerra M (2021). A novel cell-penetrating peptide targeting calpain-cleavage of PSD-95 induced by excitotoxicity improves neurological outcome after stroke. Theranostics.

[68] Duan JN, Gao SQ, Tu S, Lenahan C, Shao AW, Sheng JF (2021). Pathophysiology and therapeutic potential of NADPH oxidases in ischemic stroke-induced oxidative stress. Oxid Med Cell Longev.

[69] Espinós C, Galindo MI, García-Gimeno MA, Ibáñez-Cabellos JS, Martínez-Rubio D, Millán JM (2020). Oxidative stress, a crossroad between rare diseases and neurodegeneration. Antioxidants (Basel).

[70] Carbone F, Teixeira PC, Braunersreuther V, Mach F, Vuilleumier N, Montecucco F (2015). Pathophysiology and treatments of oxidative injury in ischemic stroke: Focus on the phagocytic NADPH oxidase 2. Antioxid Redox Signal.

[71] Shen W, Lu YG, Hu JA, Le HW, Yu W, Xu WH (2020). Mechanism of miR-320 in regulating biological characteristics of ischemic cerebral neuron by mediating Nox2/ROS pathway. J Mol Neurosci.

[72] Wu LQ, Xiong XX, Wu XM, Ye YZ, Jian ZH, Zhi Z (2020). Targeting oxidative stress and inflammation to prevent ischemia-reperfusion injury. Front Mol Neurosci.

[73] Sinha K, Das J, Pal PB, Sil PC (2013). Oxidative stress: The mitochondria-dependent and mitochondria-independent pathways of apoptosis. Arch Toxicol.

[74] Lopez J, Tait SWG (2015). Mitochondrial apoptosis: Killing cancer using the enemy within. Br J Cancer.

[75] Culmsee C, Mattson MP (2005). p53 in neuronal apoptosis. Biochem Biophys Res Commun.

[76] Kishimoto M, Suenaga J, Takase H, Araki K, Yao T, Fujimura T (2019). Oxidative stress-responsive apoptosis inducing protein (ORAIP) plays a critical role in cerebral ischemia/reperfusion injury. Sci Rep.

[77] Malko P, Jiang LH (2020). TRPM2 channel-mediated cell death: An important mechanism linking oxidative stress-inducing pathological factors to associated pathological conditions. Redox Biol.

[78] Zhang YF, Lu XY, Tai B, Li WJ, Li T (2021). Ferroptosis and its multifaceted roles in cerebral stroke. Front Cell Neurosci.

[79] Petronek MS, Spitz DR, Buettner GR, Allen BG (2019). Linking cancer metabolic dysfunction and genetic instability through the lens of iron metabolism. Cancers.

[80] Ray PD, Huang BW, Tsuji Y (2012). Reactive oxygen species (ROS) homeostasis and redox regulation in cellular signaling. Cell Signal.

[81] Liu C, Liang MC, Soong TW (2019). Nitric oxide, iron and neurodegeneration. Front Neurosci.

[82] Selim MH, Ratan RR (2004). The role of iron neurotoxicity in ischemic stroke. Ageing Res Rev.

[83] Weiland A, Wang YM, Wu WH, Lan X, Han XN, Li Q (2019). Ferroptosis and its role in diverse brain diseases. Mol Neurobiol.

[84] Yao MY, Liu T, Zhang L, Wang MJ, Yang Y, Gao J (2021). Role of ferroptosis in neurological diseases. Neurosci Lett.

[85] Wu JR, Tuo QZ, Lei P (2018). Ferroptosis, a recent defined form of critical cell death in neurological disorders. J Mol Neurosci.

[86] Yang WS, SriRamaratnam R, Welsch ME, Shimada K, Skouta R, Viswanathan VS (2014). Regulation of ferroptotic cancer cell death by GPX4. Cell.

[87] Ahmad S, Elsherbiny NM, Haque R, Khan MB, Ishrat T, Shah ZA (2014). Sesamin attenuates neurotoxicity in mouse model of ischemic brain stroke. Neurotoxicology.

[88] Zhang K, Tu MJ, Gao W, Cai XJ, Song FH, Chen Z (2019). Hollow Prussian blue nanozymes drive neuroprotection against ischemic stroke *via* attenuating oxidative stress, counteracting inflammation, and suppressing cell apoptosis. Nano Lett.

[89] Yan XL, Yu AM, Zheng HZ, Wang SX, He YY, Wang LS (2019). Calycosin-7- O- β- D-glucoside attenuates OGD/R-induced damage by preventing oxidative stress and neuronal apoptosis *via* the SIRT1/FOXO1/PGC-1 α pathway in HT22 cells. Neural Plast.

[90] Zhi SM, Fang GX, Xie XM, Liu LH, Yan J, Liu DB (2020). Melatonin reduces OGD/R-induced neuron injury by regulating redox/inflammation/apoptosis signaling. Eur Rev Med Pharmacol Sci.

[91] Liu DQ, Wang HL, Zhang YG, Zhang Z (2020). Protective effects of chlorogenic acid on cerebral ischemia/reperfusion injury rats by regulating oxidative stress-related Nrf2 pathway. Drug Des Devel Ther.

[92] Guan XY, Li XL, Yang XJ, Yan JW, Shi PL, Ba LN (2019). The neuroprotective effects of carvacrol on ischemia/reperfusion-induced hippocampal neuronal impairment by ferroptosis mitigation. Life Sci.

[93] Xu J, Wang YL, Wang AX, Gao ZQ, Gao XP, Chen HS (2019). Safety and efficacy of Edaravone Dexborneol versus edaravone for patients with acute ischaemic stroke: A phase II, multicentre, randomised, double-blind, multiple-dose, active-controlled clinical trial. Stroke Vasc Neurol.

[94] Frenguelli BG, Dale N (2020). Purines: from diagnostic biomarkers to therapeutic agents in brain injury. Neurosci Bull.

[95] Jayaraj RL, Azimullah S, Beiram R, Jalal FY, Rosenberg GA (2019). Neuroinflammation: friend and foe for ischemic stroke. J Neuroinflammation.

[96] Siracusa R, Fusco R, Cuzzocrea S (2019). Astrocytes: role and functions in brain pathologies. Front Pharmacol.

[97] Cekanaviciute E, Buckwalter MS (2016). Astrocytes: integrative regulators of neuroinflammation in stroke and other neurological diseases. Neurotherapeutics.

[98] Rosenberg GA, Estrada EY, Dencoff JE (1998). Matrix metalloproteinases and TIMPs are associated with blood-brain barrier opening after reperfusion in rat brain. Stroke.

[99] Minkiewicz J, de Rivero Vaccari JP, Keane RW (2013). Human astrocytes express a novel NLRP2 inflammasome. Glia.

[100] Božić M, Verkhratsky A, Zorec R, Stenovec M (2020). Exocytosis of large-diameter lysosomes mediates interferon γ-induced relocation of MHC class II molecules toward the surface of astrocytes. Cell Mol Life Sci.

[101] Puig B, Brenna S, Magnus T (2018). Molecular communication of a dying neuron in stroke. Int J Mol Sci.

[102] Brown GC, Neher JJ (2014). Microglial phagocytosis of live neurons. Nat Rev Neurosci.

[103] Neher JJ, Emmrich JV, Fricker M, Mander PK, Théry C, Brown GC (2013). Phagocytosis executes delayed neuronal death after focal brain ischemia. Proc Natl Acad Sci U S A.

[104] Neher JJ, Neniskyte U, Zhao JW, Bal-Price A, Tolkovsky AM, Brown GC (2011). Inhibition of microglial phagocytosis is sufficient to prevent inflammatory neuronal death. J Immunol.

[105] Allendorf DH, Puigdellívol M, Brown GC (2020). Activated microglia desialylate their surface, stimulating complement receptor 3-mediated phagocytosis of neurons. Glia.

[106] Tummers B, Green DR (2017). Caspase-8: Regulating life and death. Immunol Rev.

[107] Gilbert LC, Rubin J, Nanes MS (2005). The p55 TNF receptor mediates TNF inhibition of osteoblast differentiation independently of apoptosis. Am J Physiol Endocrinol Metab.

[108] Frank D, Vince JE (2019). Pyroptosis versus necroptosis: Similarities, differences, and crosstalk. Cell Death Differ.

[109] Yuan JY, Amin P, Ofengeim D (2019). Necroptosis and RIPK_1_-mediated neuroinflammation in CNS diseases. Nat Rev Neurosci.

[110] Cai ZY, Jitkaew S, Zhao J, Chiang HC, Choksi S, Liu J (2014). Plasma membrane translocation of trimerized MLKL protein is required for TNF-induced necroptosis. Nat Cell Biol.

[111] Seo J, Lee EW, Sung H, Seong D, Dondelinger Y, Shin J (2016). CHIP controls necroptosis through ubiquitylation- and lysosome-dependent degradation of RIPK_3_. Nat Cell Biol.

[112] Zhang S, Hu ZW, Mao CY, Shi CH, Xu YM (2020). CHIP as a therapeutic target for neurological diseases. Cell Death Dis.

[113] Cabral-Miranda F, Nicoloso-Simões E, Adão-Novaes J, Chiodo V, Hauswirth WW, Linden R (2017). rAAV8-733-mediated gene transfer of CHIP/stub-1 prevents hippocampal neuronal death in experimental brain ischemia. Mol Ther.

[114] McKenzie BA, Dixit VM, Power C (2020). Fiery cell death: Pyroptosis in the central nervous system. Trends Neurosci.

[115] Wang KK, Sun ZZ, Ru JN, Wang SM, Huang LJ, Ruan LH (2020). Ablation of GSDMD improves outcome of ischemic stroke through blocking canonical and non-canonical inflammasomes dependent pyroptosis in microglia. Front Neurol.

[116] Wang KK, Ru JN, Zhang HL, Chen JY, Lin X, Lin ZX (2020). Melatonin enhances the therapeutic effect of plasma exosomes against cerebral ischemia-induced pyroptosis through the TLR4/NF-κB pathway. Front Neurosci.

[117] Lammert CR, Frost EL, Bellinger CE, Bolte AC, McKee CA, Hurt ME (2020). AIM2 inflammasome surveillance of DNA damage shapes neurodevelopment. Nature.

[118] Zhang ZF, Zou XX, Zhang R, Xie Y, Feng ZM, Li F (2021). Human umbilical cord mesenchymal stem cell-derived exosomal miR-146a-5p reduces microglial-mediated neuroinflammation *via* suppression of the IRAK1/TRAF_6_ signaling pathway after ischemic stroke. Aging.

[119] Wang N, Liu Y, Jia CX, Gao CW, Zheng T, Wu MX (2021). Machine learning enables discovery of Gentianine targeting TLR4/NF-κB pathway to repair ischemic stroke injury. Pharmacol Res.

[120] Pan YL, Jiao QZ, Wei W, Zheng TY, Yang XY, Xin WQ (2021). Emerging role of LncRNAs in ischemic stroke-novel insights into the regulation of inflammation. J Inflamm Res.

[121] Zhang S, Sun WC, Liang ZD, Yin XR, Ji ZR, Chen XH (2020). LncRNA SNHG4 attenuates inflammatory responses by sponging miR-449c-5p and up-regulating STAT6 in microglial during cerebral ischemia-reperfusion injury. Drug Des Devel Ther.

[122] Li TH, Luo YR, Zhang P, Guo SW, Sun HW, Yan DM (1985). LncRNA MEG3 regulates microglial polarization through KLF_4_ to affect cerebral ischemia-reperfusion injury. J Appl Physiol.

[123] Jin F, Ou WY, Wei BY, Fan HY, Wei CC, Fang DZ (2021). Transcriptome-wide analysis to identify the inflammatory role of lncRNA Neat1 in experimental ischemic stroke. J Inflamm Res.

[124] Ni XR, Su Q, Xia WB, Zhang YL, Jia KJ, Su ZQ (2020). Knockdown lncRNA NEAT1 regulates the activation of microglia and reduces AKT signaling and neuronal apoptosis after cerebral ischemic reperfusion. Sci Rep.

[125] Dhanesha N, Patel RB, Doddapattar P, Ghatge M, Flora GD, Jain M (2022). PKM2 promotes neutrophil activation and cerebral thromboinflammation: Therapeutic implications for ischemic stroke. Blood.

[126] Meng HL, Zhao HR, Cao X, Hao JW, Zhang H, Liu Y (2019). Double-negative T cells remarkably promote neuroinflammation after ischemic stroke. Proc Natl Acad Sci USA.

[127] Weitbrecht L, Berchtold D, Zhang T, Jagdmann S, Dames C, Winek K (2021). CD4+ T cells promote delayed B cell responses in the ischemic brain after experimental stroke. Brain Behav Immun.

[128] Nakajima S, Tanaka R, Yamashiro K, Chiba A, Noto D, Inaba T (2021). Mucosal-associated invariant T cells are involved in acute ischemic stroke by regulating neuroinflammation. J Am Heart Assoc.

[129] Xia YG, Hu GW, Chen Y, Yuan J, Zhang JT, Wang SF (2021). Embryonic stem cell derived small extracellular vesicles modulate regulatory T cells to protect against ischemic stroke. ACS Nano.

[130] Cai HX, Ma YY, Jiang L, Mu ZH, Jiang Z, Chen XY (2017). Hypoxia response element-regulated MMP-9 promotes neurological recovery *via* glial scar degradation and angiogenesis in delayed stroke. Mol Ther.

[131] Yuan JC, Li LL, Yang QH, Ran H, Wang J, Hu KY (2021). Targeted treatment of ischemic stroke by bioactive nanoparticle-derived reactive oxygen species responsive and inflammation-resolving nanotherapies. ACS Nano.

[132] Lin SY, Wang YY, Chang CY, Wu CC, Chen WY, Liao SL (2021). TNF-α receptor inhibitor alleviates metabolic and inflammatory changes in a rat model of ischemic stroke. Antioxidants.

[133] Guo TZ, Liu YT, Ren XL, Wang W, Liu HR (2020). Promoting role of long non-coding RNA small nucleolar RNA host gene 15 (*SNHG15*) in neuronal injury following ischemic stroke *via* the microRNA-18a/CXC chemokine ligand 13 (*CXCL13*)/*ERK*/*MEK* axis. Med Sci Monit.

[134] Smith CJ, Hulme S, Vail A, Heal C, Parry-Jones AR, Scarth S (2018). SCIL-STROKE (subcutaneous interleukin-1 receptor antagonist in ischemic stroke): A randomized controlled phase 2 trial. Stroke.

[135] Deter RL, Baudhuin P, de Duve C (1967). Participation of lysosomes in cellular autophagy induced in rat liver by glucagon. J Cell Biol.

[136] Dikic I, Elazar Z (2018). Mechanism and medical implications of mammalian autophagy. Nat Rev Mol Cell Biol.

[137] Mizushima N, Levine B (2020). Autophagy in human diseases. N Engl J Med.

[138] Yu L, Chen Y, Tooze SA (2018). Autophagy pathway: Cellular and molecular mechanisms. Autophagy.

[139] Galluzzi L, Bravo-San Pedro JM, Blomgren K, Kroemer G (2016). Autophagy in acute brain injury. Nat Rev Neurosci.

[140] Tooze SA, Dikic I (2016). Autophagy captures the Nobel prize. Cell.

[141] Galluzzi L, Vitale I, Aaronson SA, Abrams JM, Adam D, Agostinis P (2018). Molecular mechanisms of cell death: Recommendations of the Nomenclature Committee on Cell Death 2018. Cell Death Differ.

[142] Kriel J, Loos B (2019). The good, the bad and the autophagosome: Exploring unanswered questions of autophagy-dependent cell death. Cell Death Differ.

[143] Denton D, Kumar S (2019). Autophagy-dependent cell death. Cell Death Differ.

[144] Liu Y, Levine B (2015). Autosis and autophagic cell death: The dark side of autophagy. Cell Death Differ.

[145] Liu Y, Shoji-Kawata S, Sumpter RM, Wei YJ, Ginet V, Zhang LY (2013). Autosis is a Na+, K+-ATPase-regulated form of cell death triggered by autophagy-inducing peptides, starvation, and hypoxia-ischemia. Proc Natl Acad Sci U S A.

[146] Cordani M, Butera G, Pacchiana R, Donadelli M (2017). Molecular interplay between mutant p53 proteins and autophagy in cancer cells. Biochim Biophys Acta Rev Cancer.

[147] Xie CC, Ginet V, Sun YY, Koike M, Zhou K, Li T (2016). Neuroprotection by selective neuronal deletion of Atg7 in neonatal brain injury. Autophagy.

[148] Zhang DM, Zhang T, Wang MM, Wang XX, Qin YY, Wu JC (2019). TIGAR alleviates ischemia/reperfusion-induced autophagy and ischemic brain injury. Free Radic Biol Med.

[149] Zhang X, Wei MP, Fan JH, Yan WJ, Zha X, Song HM (2021). Ischemia-induced upregulation of autophagy preludes dysfunctional lysosomal storage and associated synaptic impairments in neurons. Autophagy.

[150] Kim KA, Kim D, Kim JH, Shin YJ, Kim ES, Akram M, *et al*. Autophagy-mediated occludin degradation contributes to blood-brain barrier disruption during ischemia in bEnd.3 brain endothelial cells and rat ischemic stroke models. Fluids Barriers CNS 2020, 17: 21. 10.1186/s12987-020-00182-8 PMC7071658 32169114

[151] Zhou XY, Luo Y, Zhu YM, Liu ZH, Kent TA, Rong JG (2017). Inhibition of autophagy blocks cathepsins-tBid-mitochondrial apoptotic signaling pathway *via* stabilization of lysosomal membrane in ischemic astrocytes. Cell Death Dis.

[152] Su ZY, Yang ZZ, Xu YQ, Chen YB, Yu Q (2015). Apoptosis, autophagy, necroptosis, and cancer metastasis. Mol Cancer.

[153] Li Z, Chen TS, Cao YW, Jiang XX, Lin HD, Zhang J (2019). Pros and cons: Autophagy in acute spinal cord injury. Neurosci Bull.

[154] Maiuri MC, Zalckvar E, Kimchi A, Kroemer G (2007). Self-eating and self-killing: Crosstalk between autophagy and apoptosis. Nat Rev Mol Cell Biol.

[155] Huang K, O'Neill KL, Li J, Zhou W, Han N, Pang XM (2019). BH_3_-only proteins target BCL-xL/MCL-1, not BAX/BAK, to initiate apoptosis. Cell Res.

[156] Booth LA, Roberts JL, Dent P (2020). The role of cell signaling in the crosstalk between autophagy and apoptosis in the regulation of tumor cell survival in response to sorafenib and neratinib. Semin Cancer Biol.

[157] Füllgrabe J, Klionsky DJ, Joseph B (2014). The return of the nucleus: Transcriptional and epigenetic control of autophagy. Nat Rev Mol Cell Biol.

[158] Rodríguez-Hernández MA, de la Cruz-Ojeda P, López-Grueso MJ, Navarro-Villarán E, Requejo-Aguilar R, Castejón-Vega B (2020). Integrated molecular signaling involving mitochondrial dysfunction and alteration of cell metabolism induced by tyrosine kinase inhibitors in cancer. Redox Biol.

[159] Yin Y, Sun G, Li E, Kiselyov K, Sun DD (2017). ER stress and impaired autophagy flux in neuronal degeneration and brain injury. Ageing Res Rev.

[160] Bhardwaj M, Leli NM, Koumenis C, Amaravadi RK (2020). Regulation of autophagy by canonical and non-canonical ER stress responses. Semin Cancer Biol.

[161] Cai Y, Arikkath J, Yang L, Guo ML, Periyasamy P, Buch S (2016). Interplay of endoplasmic *Reticulum* stress and autophagy in neurodegenerative disorders. Autophagy.

[162] Filomeni G, de Zio D, Cecconi F (2015). Oxidative stress and autophagy: The clash between damage and metabolic needs. Cell Death Differ.

[163] Ornatowski W, Lu Q, Yegambaram M, Garcia AE, Zemskov EA, Maltepe E (2020). Complex interplay between autophagy and oxidative stress in the development of pulmonary disease. Redox Biol.

[164] Grunwald DS, Otto NM, Park JM, Song D, Kim DH (2020). GABARAPs and LC3s have opposite roles in regulating ULK1 for autophagy induction. Autophagy.

[165] Xu Y, Shen J, Ran ZH (2020). Emerging views of mitophagy in immunity and autoimmune diseases. Autophagy.

[166] Cheng ZY (2019). The FoxO-autophagy axis in health and disease. Trends Endocrinol Metab.

[167] Dikic I (2017). Proteasomal and autophagic degradation systems. Annu Rev Biochem.

[168] Kim HJ, Joe Y, Kim SK, Park SU, Park J, Chen YQ (2017). Carbon monoxide protects against hepatic steatosis in mice by inducing sestrin-2 *via* the PERK-eIF2α-ATF_4_ pathway. Free Radic Biol Med.

[169] Deretic V, Levine B (2018). Autophagy balances inflammation in innate immunity. Autophagy.

[170] Mo Y, Sun YY, Liu KY (2020). Autophagy and inflammation in ischemic stroke. Neural Regen Res.

[171] Sekerdag E, Solaroglu I, Gursoy-Ozdemir Y (2018). Cell death mechanisms in stroke and novel molecular and cellular treatment options. Curr Neuropharmacol.

[172] Amaravadi RK, Kimmelman AC, Debnath J (2019). Targeting autophagy in cancer: Recent advances and future directions. Cancer Discov.

[173] Egan DF, Chun MGH, Vamos M, Zou HX, Rong J, Miller CJ (2015). Small molecule inhibition of the autophagy kinase ULK1 and identification of ULK1 substrates. Mol Cell.

[174] Levy J, Towers CG, Thorburn A (2017). Targeting autophagy in cancer. Nat Rev Cancer.

[175] Lu Y, Wang SJ, Cai SY, Gu XX, Wang JJ, Yang Y (2020). Propofol-induced miR-20b expression initiates endogenous cellular signal changes mitigating hypoxia/re-oxygenation-induced endothelial autophagy *in vitro*. Cell Death Dis.

[176] Ajoolabady A, Wang SY, Kroemer G, Penninger JM, Uversky VN, Pratico D (2021). Targeting autophagy in ischemic stroke: From molecular mechanisms to clinical therapeutics. Pharmacol Ther.

[177] Peng F, Qiu LH, Yao MY, Liu LD, Zheng YF, Wu SL (2021). A lithium-doped surface inspires immunomodulatory functions for enhanced osteointegration through PI3K/AKT signaling axis regulation. Biomater Sci.

[178] Beaumatin F, O'Prey J, Barthet VJA, Zunino B, Parvy JP, Bachmann AM (2019). mTORC1 activation requires DRAM-1 by facilitating lysosomal amino acid efflux. Mol Cell.

[179] Levine B, Kroemer G (2019). Biological functions of autophagy genes: A disease perspective. Cell.

[180] Han B, Zhang Y, Zhang YH, Bai Y, Chen XF, Huang RR (2018). Novel insight into circular RNA HECTD1 in astrocyte activation *via* autophagy by targeting MIR142-TIPARP: Implications for cerebral ischemic stroke. Autophagy.

[181] Tang Y, Liu JC, Wang Y, Yang L, Han B, Zhang Y (2021). PARP14 inhibits microglial activation *via* LPAR5 to promote post-stroke functional recovery. Autophagy.

[182] Kim JH, Kim SY, Kim B, Lee SR, Cha SH, Lee DS (2021). Prospects of therapeutic target and directions for ischemic stroke. Pharmaceuticals (Basel).

[183] Zhou HH, Tang Y, Zhang XY, Luo CX, Gao LY, Wu HY (2015). Delayed administration of tat-HA-NR2B9c promotes recovery after stroke in rats. Stroke.

[184] Qin C, Zhou LQ, Ma XT, Hu ZW, Yang S, Chen M (2019). Dual functions of microglia in ischemic stroke. Neurosci Bull.

[185] Li Y, Liu ZW, Xin HQ, Chopp M (2014). The role of astrocytes in mediating exogenous cell-based restorative therapy for stroke. Glia.

[186] Wang P, Shao BZ, Deng ZQ, Chen S, Yue ZY, Miao CY (2018). Autophagy in ischemic stroke. Prog Neurobiol.

